# Mammalian DNA methyltransferases in DNA methylation and imprinted gene expression in extraembryonic ectoderm of post-implantation embryos

**DOI:** 10.1016/j.jbc.2026.113554

**Published:** 2026-09-11

**Authors:** Ting Lu, Yilin Li, Yuping Wen, Zhen Xu, Haodong Lin, Guofeng Shao, Xiajun Li

**Affiliations:** 1 School of Life Science and Technology, ShanghaiTech University, Shanghai, China; 2 Department of Orthopedic Surgery, Shanghai General Hospital, Shanghai Jiaotong University School of Medicine, Shanghai, China; 3 Department of Cardiovascular Surgery, The Affiliated Lihuili Hospital of Ningbo University, Ningbo City, Zhejiang, China; 4 Ningbo Key Laboratory for Innovative Research in Aortic Surgery Diagnosis and Treatment, The Affiliated Lihuili Hospital of Ningbo University, Ningbo City, Zhejiang, China

**Keywords:** DNA methylation, *de novo* methylation, maintenance methylation, DNMT3A, DNMT3B, DNMT1, genomic imprinting, imprinted gene, allelic expression switch, imprinting control region (ICR), differentially methylated region (DMR)

## Abstract

DNA methylation in mammals is mainly catalyzed by three DNA methyltransferases (DNMTs). Conventionally, DNMT1 is considered the primary DNMT protein for maintenance DNA methylation, whereas DNMT3A and DNMT3B function in *de novo* DNA methylation. In two previous studies, we demonstrated that DNMT3A and DNMT3B maintain genome-wide DNA methylation in embryonic stem (ES) cells and in the epiblast of post-implantation embryos. Interestingly, DNMT3A and DNMT3B also sustain genome-wide DNA methylation in the extraembryonic ectoderm (EXE) of post-implantation embryos, including repeats, genic and intergenic regions. Although DNMT1 plays a major role in maintaining DNA methylation at the imprinting control regions (ICRs) in the imprinted regions, DNMT3A and DNMT3B are required for preserving DNA methylation at the ICRs of a subset of imprinted regions in EXE, similar to the observations in ES cells and epiblast. Surprisingly, *de novo* DNA methylation mediated by DNMT3A and DNMT3B leads to increased DNA methylation at a large subset of imprinted regions. These results are consistent with what we previously elucidated in the epiblast of post-implantation embryos. Importantly, loss of DNA methylation at the ICR of an imprinted region, resulting from the absence of DNMT1 or two DNMT3 proteins, causes allelic expression switch of the corresponding imprinted genes in that imprinted region. This study provides further evidence that DNMT3A and DNMT3B exert both maintenance and *de novo* DNA methylation functions across the genome in post-implantation embryos. It also validates some previous findings for DNA methylation-dependent allelic expression switch of imprinted genes.

DNA methylation occurs mainly at CpG sites in mammals. It is mainly catalyzed by three mammalian DNA methyltransferase (DNMT) proteins (DNMT1, DNMT3A, and DNMT3B) in mice (1, 2). The murine DNMT3C protein, a product of gene duplication in evolution, appears to be primarily expressed in the male germ cells of mice (1, 3, 4). Conventionally, DNMT1 is essential for maintenance DNA methylation, while DNMT3A and DNMT3B are required for *de novo* DNA methylation in post-implantation embryos (5, 6).

Although DNMT1 used to be regarded as the primary DNMT responsible for maintenance DNA methylation, two DNMT3 proteins (DNMT3A and DNMT3B) have been shown to play important roles in maintaining DNA methylation across the genome in embryonic stem (ES) and post-implantation embryos (6, 7, 8). It is reported that DNMT1 may play a role in *de novo* DNA methylation, while it is primarily involved in maintaining DNA methylation during mouse embryonic development (9, 10). There was some evidence indicating that DNMT3A and DNMT3B may contribute to maintenance DNA methylation in mouse ES cells (11, 12). Interestingly, DNMT3A and DNMT3B maintain DNA methylation, together with DNMT1, across the genome, including repeats, genic and intergenic regions in mouse ES cells according to our previous study (8). Recently, we found that DNMT3A and DNMT3B are critical for genome-wide DNA methylation maintenance in the epiblast of post-implantation embryos, besides their well-known roles in *de novo* DNA methylation (6).

Genomic imprinting is characterized by parent-of-origin-dependent mono-allelic expression of a few hundred imprinted genes in eutherian mammals (13, 14, 15). Most of the roughly 200 known imprinted genes in mice are clustered in 24 known imprinted regions (16, 17, 18). Expression of the clustered imprinted genes is co-regulated by a cis-acting imprinting control region (ICR) carrying a differentially methylated region (DMR) with germline-derived DNA methylation (19, 20). Differential DNA methylation at the ICR is established during oogenesis or spermatogenesis, and it plays critical roles in regulating mono-allelic expression of the corresponding imprinted genes in that imprinted region (16, 21, 22). DNA methylation at most known ICRs is established during oogenesis and therefore resides at the ICRs on the maternal chromosomes, whereas DNA methylation at three ICRs (*H19*, *Rasgrf1* and IG-DMR of the *Dlk1*-*Meg3* imprinted region) is acquired during spermatogenesis and thus inherited at the ICRs on the paternal chromosomes (21, 23). Differential DNA methylation reconstituted at the ICRs after fertilization is maintained thereafter during embryogenesis, mainly through ZFP57-dependent recruitment of DNMTs to the ICRs *via* KAP1/Trim28 (24, 25, 26, 27, 28). Indeed, ZFP57 maintains differential DNA methylation at the ICRs of most known imprinted regions in mouse embryos and embryonic stem (ES) cells (29, 30). Previously, we demonstrated that ZFP57 dictates the allelic expression switch of the target imprinted genes in mouse embryos through maintenance of DNA methylation at the ICRs of the corresponding imprinted regions (30). Interestingly, DNMT3A and DNMT3B contribute to the maintenance of DNA methylation at a subset of ICRs in ES cells and post-implantation embryos (6, 8).

Genomic imprinting is essential for both embryonic and extraembryonic development (31, 32, 33). It is still unclear how DNMT1, DNMT3A, and DNMT3B may regulate extraembryonic development and parent-of-origin-dependent monoallelic expression of imprinted genes. In this study, we have obtained similar results implicating the important roles of DNMT3A and DNMT3B in maintaining DNA methylation across the genome in the extraembryonic ectoderm (EXE) of post-implantation embryos, including DNA methylation at the repeats, retroviral elements, genic and intergenic regions as well as DNA methylation at the ICRs in a subset of imprinted regions. As expected, DNMT3A and DNMT3B are also required for *de novo* DNA methylation in EXE after implantation. In addition, loss of DNA methylation at the ICR of an imprinted region in the absence of DNMT1 or two DNMT3 proteins causes an allelic expression switch of the corresponding imprinted genes in that imprinted region. Besides DNMT1, DNMT3A and DNMT3B also contribute to the maintenance of monoallelic expression of some imprinted genes according to our current study.

## Results

### DNA methylation in the genic and intergenic regions

DNA methylation data were downloaded from two published studies for the trophectoderm (TE) of the pre-implantation E3.5 embryos and for the extraembryonic ectoderm (EXE) of the E6.5 or E7.5 post-implantation embryos (34, 35). DNMTs were analyzed for their functions in whole-genome DNA methylation in TE at E3.5, and in EXE at E6.5 or E7.5 (Fig. 1, *A* and *B*). Just under 20% DNA methylation was obtained when all CpG sites were quantified for their average DNA methylation levels in TE of the E3.5 pre-implantation embryos (Fig. 1, *A* and *B*) (35). After implantation, DNA methylation increased to about 50% in EXE at E6.5 and further increased to roughly 60% in EXE at E7.5 (Fig. 1, *A* and *B*). The increased DNA methylation was eliminated in EXE of the *Dnmt3* DKO mutant post-implantation embryos at E6.5 or E7.5 lacking *Dnmt3a* and *Dnmt3b*, indicating the roles of *de novo* DNA methylation mediated by DNMT3A and DNMT3B that were responsible for increase in DNA methylation in EXE of WT E6.5-E7.5 embryos (Fig. 1, *A* and *B*). As expected, DNA methylation was completely missing in EXE of *Dnmt* TKO mutant embryos at E6.5 or E7.5 without *Dnmt1*, *Dnmt3a*, or *Dnmt3b* (Fig. 1, *A* and *B*). In contrast, DNA methylation was partially lost in EXE of *Dnmt1* KO or *Dnmt3* DKO mutant embryos at E6.5 and E7.5 (Fig. 1, *A* and *B*).

**Figure 1:**
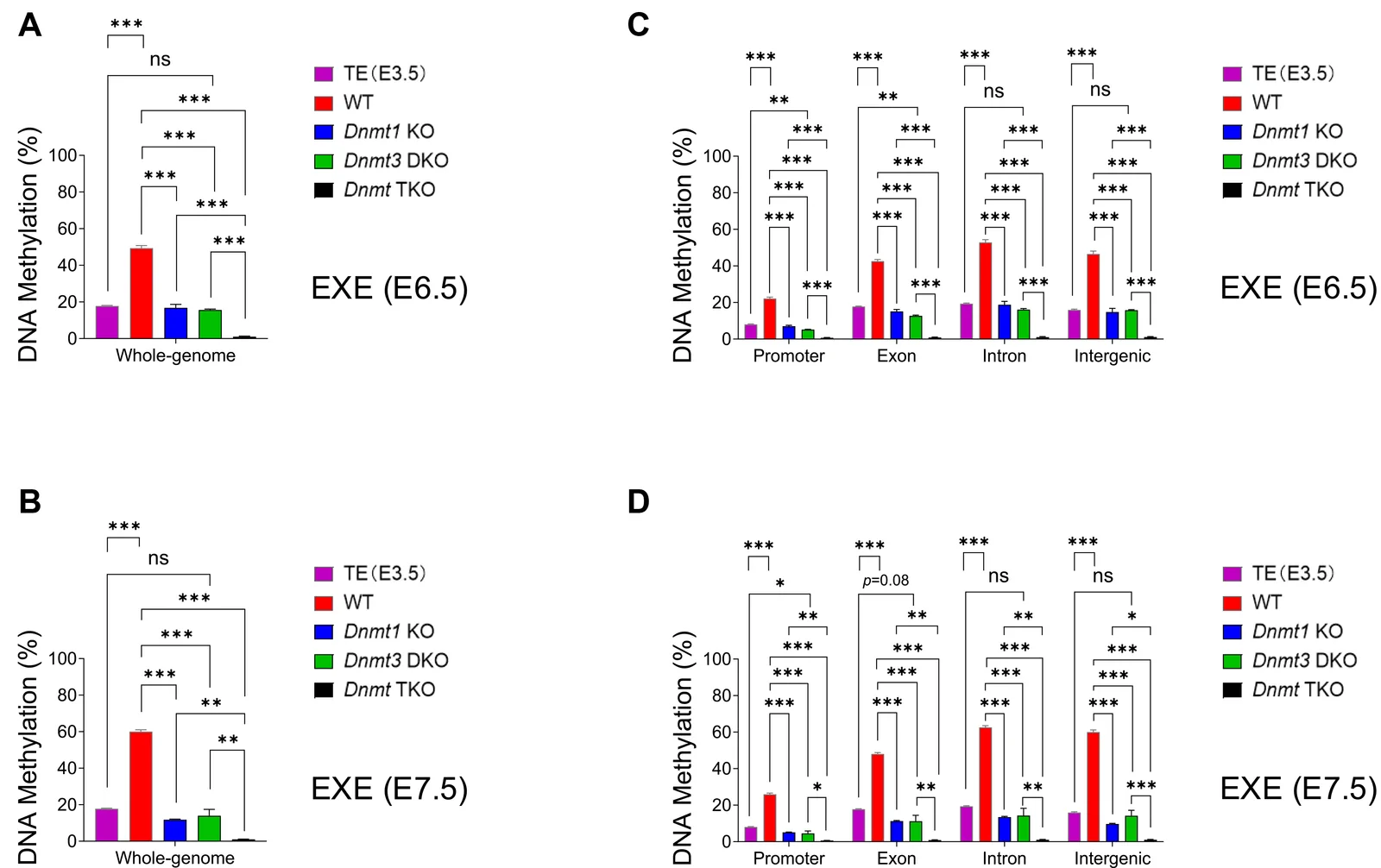
**Three DNMT proteins were complementary in maintaining whole-genome DNA methylation as well as DNA methylation in the genic and intergenic regions in the extraembryonic ectoderm of post-implantation embryos.** DNA methylation was quantified and compared for the trophectoderm (TE) of the wild-type (WT) E3.5 embryos, the extraembryonic ectoderm (EXE) of WT and *Dnmt* mutant E6.5 and E7.5 embryos. Horizontal axis, the analyzed DNA samples from TE of WT E3.5 embryos, and from EXE of WT, *Dnmt1* mutant (*Dnmt1* KO), *Dnmt3* double mutant (*Dnmt3* DKO) and *Dnmt* triple mutant (*Dnmt* TKO) E6.5 or E7.5 embryos. Vertical axis, % of DNA methylation. DNA methylation level was compared by using one-way ANOVA with Dunnett multiple comparison test. The statistical analysis results are shown on the figures as follows: ∗, *p* < 0.05; ∗∗, *p* < 0.01; ∗∗∗, *p* < 0.001. ns, not statistically significant, with a *p*-value over 0.1. *A*, whole-genome DNA methylation was analyzed for TE of WT E3.5 embryos, and for EXE of WT, *Dnmt1* KO, *Dnmt3* DKO and *Dnmt* TKO E6.5 embryos. *B*, whole-genome DNA methylation was analyzed for TE of WT E3.5 embryos, and for EXE of WT, *Dnmt1* KO, *Dnmt3* DKO and *Dnmt* TKO E7.5 embryos. *C*, DNA methylation was analyzed for the genic and intergenic regions in the WT TE samples, the EXE samples of WT, *Dnmt1* mutant (*Dnmt1* KO), *Dnmt3* double mutant (*Dnmt3* DKO) and *Dnmt* triple mutant (*Dnmt* TKO) E6.5 embryos. *D*, DNA methylation was analyzed for the genic and intergenic regions in the WT TE samples, the EXE samples of WT, *Dnmt1* mutant (*Dnmt1* KO), *Dnmt3* double mutant (*Dnmt3* DKO) and *Dnmt* triple mutant (*Dnmt* TKO) E7.5 embryos.

To gain further insight into changes in DNA methylation in the genic and intergenic regions, we analyzed DNA methylation in the promoters, exons and introns for the genic regions as well as in the intergenic regions in TE of the E3.5 pre-implantation embryos and in EXE of E6.5-E7.5 post-implantation embryos (Fig. 1, *C* and *D*). About 20% DNA methylation was observed in the exons and introns of TE of WT E3.5 embryos and in the intergenic regions of TE of WT E3.5 embryos (Fig. 1, *C* and *D*). DNA methylation increased to more than 40% in the intergenic regions and around 50% in the introns in EXE of WT E6.5, and it further increased to around 60% in the intergenic regions and in the introns in EXE of WT E7.5 embryos (Fig. 1, *C* and *D*). This increase in DNA methylation was abolished in the intergenic regions and in the introns in EXE of *Dnmt3* DKO mutant embryos at E6.5 or at E7.5, indicating that *de novo* DNA methylation mediated by DNMT3 proteins accounts for the increased DNA methylation in the intergenic regions and in the introns in EXE after implantation (Fig. 1, *C* and *D*). DNA methylation increased to roughly 40% in the exons in EXE of WT E6.5 and further increased to close to 50% DNA methylation in the exons in EXE of WT E7.5 embryos (Fig. 1, *C* and *D*). Interestingly, there was a significant reduction in DNA methylation in the exons when comparing EXE of *Dnmt3* DKO mutant embryos with TE of WT E3.5 embryos (Fig. 1, *C* and *D*). These results suggest that two DNMT3 proteins are involved in maintaining preexisting DNA methylation in the exons in EXE after implantation, besides their known *de novo* DNA methyltransferase activities responsible for adding new DNA methylation in the exons in EXE after implantation (Fig. 1, *C* and *D*).

Less than 10% DNA methylation was observed in the promoter regions in TE of WT E3.5 embryos (Fig. 1, *C* and *D*). It increased to more than 20% in EXE of WT E6.5 embryos and further increased to around 25% in EXE of WT E7.5 embryos (Fig. 1, *C* and *D*). Interestingly, there was also a significant reduction in DNA methylation in the promoters when comparing EXE of *Dnmt3* DKO mutant embryos at E6.5 or E7.5 with TE of WT E3.5 embryos (Fig. 1, *C* and *D*). These results suggest that two DNMT3 proteins contribute to the maintenance of pre-existing DNA methylation in the promoters in EXE after implantation. They are also responsible for *de novo* DNA methylation in the promoters in EXE after implantation (Fig. 1, *C* and *D*).

DNA methylation in three separate intergenic regions was closely analyzed to test whether three DNMT proteins may play redundant roles in maintaining DNA methylation in these genomic regions (Table S1) (36). Around 10% of DNA methylation was observed in Intergenic one and Intergenic two in the TE of WT E3.5 embryos, whereas more than 20% of the CpG sites were methylated in Intergenic three in the TE of WT E3.5 embryos (Fig. S1, *A* and *B*). DNA methylation increased to around 40% in these three intergenic regions in EXE of WT E6.5 embryos, and it further increased to around 60% in these intergenic regions in EXE of WT E7.5 embryos (Fig. S1, *A* and *B*). This increase in DNA methylation in three intergenic regions in EXE after implantation was abolished in EXE of *Dnmt3* DKO mutant E6.5-E7.5 embryos, suggesting that *de novo* DNA methylation mediated by DNMT3A and DNMT3B accounts for the increased DNA methylation (Fig. S1). A significant amount of DNA methylation remained on three intergenic regions in EXE of both *Dnmt1* KO and *Dnmt3* DKO mutant E6.5 or E7.5 embryos compared with EXE of *Dnmt* TKO mutant E6.5 or E7.5 embryos, although DNA methylation was absent in EXE of *Dnmt* TKO mutant E6.5 or E7.5 embryos (Fig. S1, *A* and *B*). These results indicate that three DNMT proteins play complementary roles in maintaining DNA methylation in these three intergenic regions in EXE after implantation.

These findings are similarly obtained when DNA methylation at the individual CpG sites of three intergenic regions was visualized on the IGV plots for DNA methylation in TE of E3.5 embryos and in EXE of E6.5-E7.5 embryos (Fig. S2). DNA methylation increased at the CpG sites across the entire Intergenic region one in EXE of WT E6.5 and E7.5 embryos in comparison to TE of two independent WT E3.5 embryos (Fig. S2*A*). This increase in DNA methylation was eliminated at the CpG sites across this entire region in EXE of *Dnmt3* DKO mutant E6.5-E7.5 embryos (Fig. S2*A*). Partial loss of DNA methylation was observed at the CpG sites across this region in EXE of both *Dnmt1* KO and *Dnmt3* DKO mutant E6.5 or E7.5 embryos, whereas DNA methylation was only observed at limited CpG sites across this region in EXE of *Dnmt* TKO mutant E6.5 or E7.5 embryos (Fig. S2*A*). Similar results were obtained from the IGV plots for DNA methylation at the CpG sites across Intergenic region two and Intergenic region 3 (Fig. S2, *B* and *C*).

In summary, *de novo* DNA methylation mediated by DNMT3A and DNMT3B causes increased DNA methylation in the intergenic and genic regions, including promoters, exons, and introns in EXE of WT embryos after implantation. Two DNMT3 proteins, together with DNMT1, maintain DNA methylation in the intergenic and genic regions in EXE after implantation. Furthermore, DNMT3A and DNMT3B contribute to the maintenance of pre-existing DNA methylation present in the promoters and exons in EXE after implantation.

### DNA methylation in the repeats and retroviral elements

Similar findings were observed when DNA methylation was examined in the repeats and retroviral elements in EXE of E6.5 or E7.5 embryos. Except for low levels of DNA methylation (roughly 10%) in the Low Complexity regions, about 20% of DNA methylation was observed in these genomic regions of TE of E3.5 embryos that include DNA repeat, LINE, SINE, LTR, Simple repeat and Satellite repeat regions (Fig. 2). DNA methylation increased to 50% or more in most repeats (DNA repeat, LINE, SINE, LTR and Satellite repeat) in EXE of WT E6.5 embryos and 60% or more in EXE of WT E7.5 embryos (Fig. 2, *A* and *B*). The increased DNA methylation was eliminated in EXE of the E6.5 or E7.5 *Dnmt3* DKO mutant embryos and largely reduced in EXE of the *Dnmt1* KO mutant E6.5 or E7.5 embryos (Fig. 2, *A* and *B*). DNA methylation increased to about 40% in the Simple repeat regions in EXE of WT E6.5 embryos and to around 50% in EXE of WT E7.5 embryos (Fig. 2, *A* and *B*). This increased DNA methylation was abolished and further reduced in EXE of the *Dnmt3* DKO mutant embryos (Fig. 2, *A* and *B*). These results suggest that *de novo* DNA methylation mediated by DNMT3A and DNMT3B accounts for the increased DNA methylation, and two DNMT3 proteins also contribute to the maintenance of pre-existing DNA methylation in the Simple repeat regions in EXE after implantation. There was no increase in DNA methylation in the low-complexity regions in EXE of WT E6.5 or E7.5 embryos (Fig. 2, *A* and *B*). However, DNA methylation was both significantly lost in the Low complexity regions in EXE of *Dnmt1* KO or *Dnmt3* DKO mutant embryos at E6.5 or E7.5, although it was still significantly higher in EXE of *Dnmt1* KO or *Dnmt3* DKO mutant E6.5-E7.5 embryos in comparison to *Dnmt* TKO mutant E6.5-E7.5 embryos (Fig. 2). These results suggest that DNMT3A and DNMT3B, together with DNMT1, maintain pre-existing DNA methylation in the Low complexity regions in EXE after implantation.

**Figure 2:**
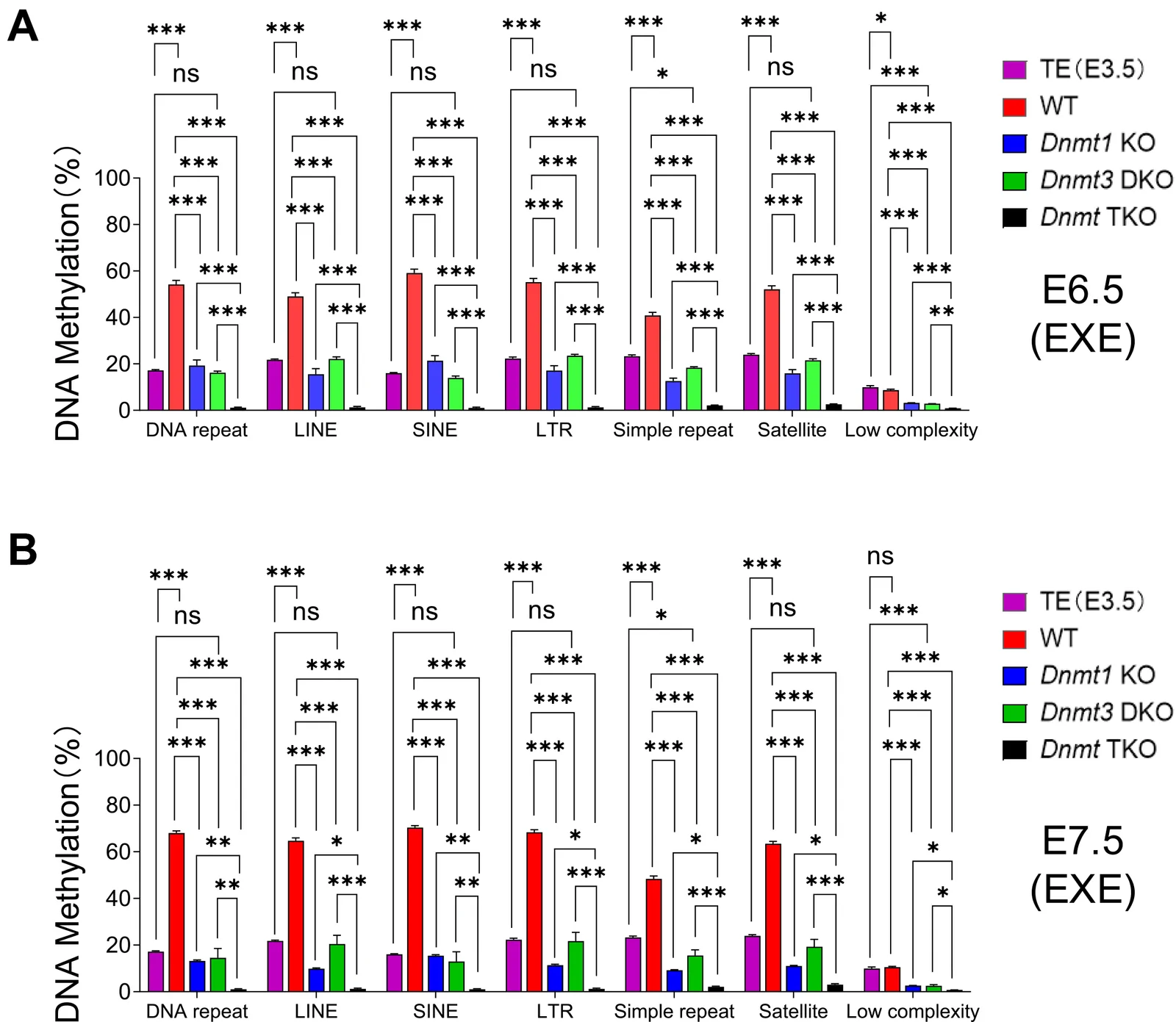
**Three DNMT proteins were complementary in maintaining DNA methylation in the repeats in the extraembryonic ectoderm of post-implantation embryos.** DNA methylation was examined for the repeats in the trophectoderm (TE) of the wild-type (WT) E3.5 embryos, the extraembryonic ectoderm (EXE) of WT and *Dnmt* mutant E6.5 and E7.5 embryos. Horizontal axis, the analyzed repeats that include DNA repeat, LINE, SINE and LTR repeats, Simple repeat, Satellite repeat, and low-complexity repeat regions. Vertical axis, % of DNA methylation. *A*, DNA methylation was analyzed for DNA repeat, LINE, SINE, LTR, Simple repeat, Satellite, and Low complexity repeat regions in the WT TE samples, the EXE samples of WT, *Dnmt1* mutant (*Dnmt1* KO), *Dnmt3* double mutant (*Dnmt3* DKO) and *Dnmt* triple mutant (*Dnmt* TKO) E6.5 embryos. *B*, DNA methylation was analyzed for DNA repeat, LINE, SINE, LTR, Simple repeat, Satellite, and Low complexity repeat regions in the WT TE samples, the EXE samples of WT, *Dnmt1* mutant (*Dnmt1* KO), *Dnmt3* double mutant (*Dnmt3* DKO) and *Dnmt* triple mutant (*Dnmt* TKO) E7.5 embryos. DNA methylation levels in the repeat regions were compared by using one-way ANOVA with Dunnett multiple comparison test. The obtained statistical analysis results are shown on the figures as follows: ∗, *p* < 0.05; ∗∗, *p* < 0.01; ∗∗∗, *p* < 0.001. ns, not statistically significant, with a *p*-value greater than 0.1.

Taken together, *de novo* DNA methylation mediated by DNMT3A and DNMT3B accounts for the increased DNA methylation observed in most repeat regions in EXE after implantation, except for no increased DNA methylation in the low-complexity regions in EXE at E6.5 or E7.5. DNMT3A and DNMT3B, together with DNMT1, maintain DNA methylation in all examined repeats in EXE after implantation. Furthermore, they contribute to the maintenance of pre-existing DNA methylation in the Simple repeat and low-complexity regions in EXE after implantation.

### DNA methylation in the CpG islands and marker genes

There was only a low level of DNA methylation (less than 4%) observed in the CpG island (CGI) regions in TE of WT E3.5 embryos (Fig. S3). DNA methylation increased to close to 6% at the CGIs in EXE of WT E6.5-E7.5 embryos (Fig. S3, *A* and *B*). DNA methylation was partially lost in EXE of *Dnmt1* KO or *Dnmt3* DKO mutant E6.5-E7.5 embryos in comparison to the WT E6.5-E7.5 embryos, whereas DNA methylation was completely lost in EXE of *Dnmt* TKO mutant E6.5-E7.5 embryos (Fig. S3, *A* and *B*). These results suggest that three DNMT proteins maintain DNA methylation at the CGIs in EXE of post-implantation embryos. Interestingly, DNA methylation was significantly reduced at the CGIs in EXE of *Dnmt1* KO or *Dnmt3* DKO mutant E6.5-E7.5 embryos in comparison to TE of WT E3.5 embryos (Fig. S3, *A* and *B*). These results indicate that DNMT3A and DNMT3B, together with DNMT1, maintain the pre-existing DNA methylation at the CGIs in EXE after implantation, which is similar to what was found in the epiblast of post-implantation embryos (6).

DNA methylation was analyzed for *Oct4* and *Nanog*, two pluripotent marker genes expressed in the inner cell mass of E3.5 embryos, in TE of WT E3.5 embryos and in EXE of E6.5-E7.5 embryos (Fig. S4). As expected, little DNA methylation was present on *Oct4* and *Nanog* in TE of WT E3.5 embryos (Fig. S4). De novo DNA methylation occurred at these two genes in EXE after implantation (Table S2) (Fig. S4). Roughly 50% DNA methylation was observed in the *Oct4* gene in EXE of WT E6.5 or E7.5 embryos, whereas around 70% DNA methylation was obtained for the CpG sites in the *Nanog* gene in EXE of WT E6.5 or E7.5 embryos (Fig. S4, *A* and *B*). This increase in DNA methylation was abolished at *Oct4* and *Nanog* in EXE of *Dnmt3* DKO mutant E6.5-E7.5 embryos (Fig. S4), indicating that two DNMT3 proteins were responsible for *de novo* DNA methylation occurring at these two genes in EXE of the post-implantation embryos.

DNA methylation was almost completely lost at *Oct4* and *Nanog* in EXE of *Dnmt* TKO mutant E6.5-E7.5 embryos (Fig. S4, *A* and *B*). DNA methylation was significantly higher at both *Oct4* and *Nanog* in EXE of *Dnmt1* KO or *Dnmt3* DKO E6.5 embryos, and at *Nanog* in EXE of *Dnmt1* KO E7.5 embryos in comparison to the corresponding *Dnmt* TKO mutant embryos (Fig. S4, *A* and *B*). DNA methylation was also significantly higher at *Nanog* in EXE of *Dnmt1* KO mutant E6.5-E7.5 embryos compared with that of *Dnmt3* DKO mutant E6.5-E7.5 embryos (Fig. S4, *A* and *B*). These results indicate the functions of DNMT3A and DNMT3B in maintaining newly acquired DNA methylation at the *Nanog* gene in EXE after implantation. These conclusions are further supported by visualizing DNA methylation of individual CpG sites at these two pluripotent marker genes on the IGV plots (Fig. S4, *C* and *D*).

At the stage of E3.5 embryos, *Gata4* and *Gata6* are expressed in the primitive endoderm, whereas *Cdx2* is expressed in TE. To gain further insights into the functions of three DNMT proteins in cell lineage differentiation of EXE after implantation, DNA methylation was examined for *Gata4*, *Gata6*, and *Cdx2* in EXE of E6.5-E7.5 embryos (Table S2) (Fig. S5). More than 10% of CpG sites were methylated at *Gata4* in TE of E3.5 embryos, whereas there was less than 5% of DNA methylation at *Gata6* and little DNA methylation at *Cdx2* in TE of WT E3.5 embryos (Fig. S5, *A* and *B*). DNA methylation increased to slightly over 30% at *Gata4* in EXE of WT E6.5 embryos and it further increased to close to 35% at *Gata4* in EXE of WT E7.5 embryos. Similarly, DNA methylation increased to around 30% at *Gata6* in EXE of WT E6.5-E7.5 embryos. There was also increased DNA methylation at *Cdx2* in EXE of WT E6.5-E7.5 embryos, with 5% to 10% of DNA methylation observed at *Cdx2* compared with little DNA methylation in TE of WT E3.5 embryos. This gain of DNA methylation was eliminated at these three genes in EXE of *Dnmt3* DKO mutant E6.5-E7.5 embryos. Interestingly, DNA methylation was further reduced at *Gata4* in EXE of *Dnmt3* DKO mutant E6.5-E7.5 embryos, compared with TE of WT E3.5 embryos (Fig. S5, *A* and *B*). Partial loss of DNA methylation was observed in these three genes in EXE of *Dnmt1* KO mutant E6.5-E7.5 embryos, whereas DNA methylation was completely missing in EXE of *Dnmt* TKO mutant E6.5 or E7.5 embryos. These results suggest that three DNMT proteins play complementary roles in maintaining DNA methylation at these three genes in EXE after implantation. Furthermore, DNMT3A and DNMT3B contribute to the maintenance of preexisting DNA methylation present at *Gata4* in EXE after implantation. Similar results were obtained when DNA methylation at the individual CpG sites was visualized at these three genes on the IGV plots for DNA methylation in TE of WT E3.5 embryos and in EXE of E6.5-E7.5 embryos (Fig. S5, *C*–*E*).

In summary, two DNMT3 proteins are responsible for increased DNA methylation at CGIs and marker genes in EXE after implantation. They contribute to maintenance of both newly acquired and pre-existing DNA methylation at CGIs and marker genes.

### DNA methylation in over half imprinted regions is primarily maintained by DNMT1 in EXE of mouse embryos

Currently, there are 24 known imprinted regions identified in mice (Table S3) (6, 37). DNA methylation at the ICRs of these imprinted regions was analyzed for TE of WT E3.5 embryos and for EXE of WT and *Dnmt* mutant E6.5-E7.5 embryos (Tables S4–S6) (Figure 3, Figure 4, Figure 5). Similar levels of DNA methylation were observed at 14 ICRs in the TE of WT E3.5 embryos and in EXE of WT E6.5-E7.5 embryos (Fig. 3, *A* and *B*). DNA methylation decreased in these 14 ICRs in the *Dnmt1* KO mutant E6.5-E7.5 embryos compared with WT E6.5-E7.5 embryos (Fig. 3, *A* and *B*). DNA methylation was absent in these ICRs in EXE of *Dnmt* TKO mutant E6.5-E7.5 embryos, as expected (Tables S5 and S6) (Fig. 3, *A* and *B*). Compared with EXE of WT E6.5 embryos, DNA methylation was not significantly reduced at these 14 ICRs in EXE of *Dnmt3* DKO mutant E6.5 embryos (Fig. 3, *A* and *B*). DNA methylation was not significantly reduced at 13 ICRs in EXE of *Dnmt3* DKO mutant E7.5 embryos either, although it was significantly reduced at the *Nap1l5* ICR in EXE of E7.5 *Dnmt3* DKO mutant embryos in comparison to EXE of WT E7.5 embryos, which may require further investigation (Fig. 3, *A* and *B*).

**Figure 3:**
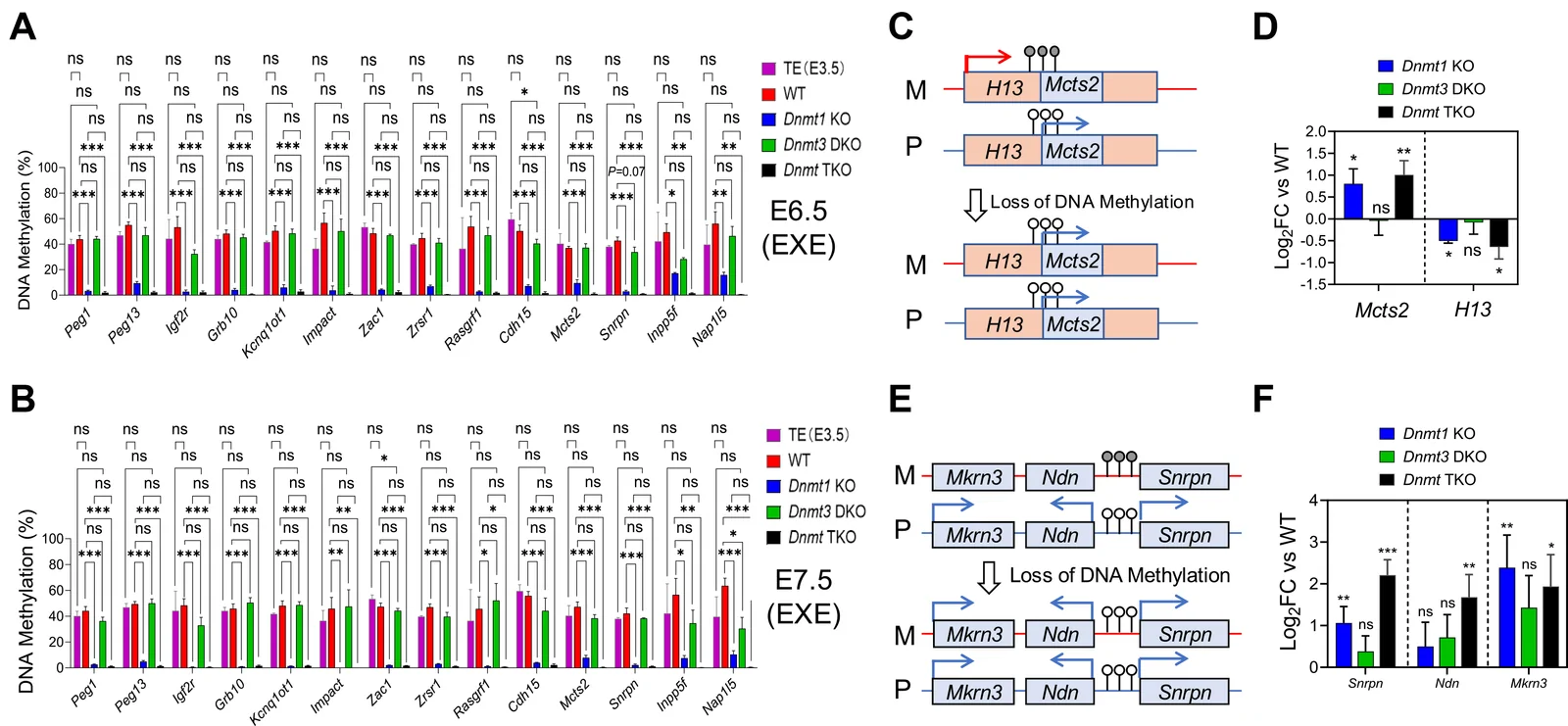
**DNMT1 maintained DNA methylation and imprinted gene expression in most imprinted regions in the extraembryonic ectoderm of the post-implantation embryos.** DNA methylation in the imprinting control region (ICR) of 24 known imprinted regions was examined for the genomic DNA samples derived from the trophectoderm (TE) of the wild-type (WT) E3.5 embryos, the extraembryonic ectoderm (EXE) of WT and *Dnmt* mutant E6.5 and E7.5 embryos. DNA methylation in 14 ICRs was statistically significantly lost in the EXE of *Dnmt1* KO, but not in the EXE of *Dnmt3* DKO, mutant E6.5-E7.5 embryos. These ICRs include the ICRs of *Peg1, Peg13, Igf2r, Grb10, Kcnq1ot1, Impact, Zac1, Zrsr1, Rasgrf1, Cdh15, Mcts2, Snrpn, Inpp5f* and *Nap1l5* imprinted regions. Expression of the corresponding imprinted genes was also analyzed for these imprinted regions in the extraembryonic ectoderm (EXE) of WT and *Dnmt* mutant E6.5 and E7.5 embryos. *A*, DNA methylation was analyzed for 14 ICRs in the WT TE, the EXE of WT, *Dnmt1* mutant (*Dnmt1* KO), *Dnmt3* double mutant (*Dnmt3* DKO) and *Dnmt* triple mutant (*Dnmt* TKO) E6.5 embryos. *B*, DNA methylation was analyzed for 14 ICRs in the WT TE, the EXE of WT, *Dnmt1* mutant (*Dnmt1* KO), *Dnmt3* double mutant (*Dnmt3* DKO) and *Dnmt* triple mutant (*Dnmt* TKO) E7.5 embryos. *C*, the diagrams are shown to illustrate expression of the *H13* and *Mcts2* imprinted genes upon loss of DNA methylation at the ICR in the *Mcts2* imprinted region according to the allelic expression switch model. Filled lollipops, methylated CpG sites in the ICR. Unfilled lollipops, unmethylated CpG sites in the ICR. *D*, relative expression levels of the *H13* and *Mcts2* imprinted genes were determined by DESeq2 with default settings comparing gene expression levels in the EXE samples of *Dnmt1* KO, *Dnmt3* DKO or *Dnmt* TKO mutant E7.5 embryos to the EXE samples of WT E7.5 embryos. One potential outlier with an exceptionally high value was excluded from the expression analysis for *H13* (see Table S7). Vertical axis, log_2_ fold change (log_2_FC) obtained from DESeq2 for relative expression levels of *H13* and *Mcts2* comparing *Dnmt* mutant EXE samples to WT EXE samples. *E*, the diagrams illustrate expression of the *Snrpn, Ndn* and *Mkrn3* imprinted genes upon loss of DNA methylation at the ICR in the *Snrpn* imprinted region according to the allelic expression switch model. Filled lollipops, methylated CpG sites in the ICR. Unfilled lollipops, unmethylated CpG sites in the ICR. *F*, relative expression levels of the *Snrpn, Ndn* and *Mkrn3* imprinted genes were determined by DESeq2 with default settings comparing gene expression levels in the EXE samples of *Dnmt1* KO, *Dnmt3* DKO or *Dnmt* TKO mutant E7.5 embryos to the EXE samples of WT E7.5 embryos. The expression levels of *Ndn* and *Mkrn3* were relatively low with high variability, and therefore *Ndn* expression was not significantly increased in *Dnmt1* KO in comparison to the EXE samples of WT E7.5 embryos although *Mkrn3* expression appeared to be significantly higher in *Dnmt1* KO compared with that in the EXE samples of WT E7.5 embryos (see Table S7). Vertical axis, log_2_ fold change (log_2_FC) obtained from DESeq2 for relative expression levels of *Snrpn, Ndn* and *Mkrn3* comparing *Dnmt* mutant EXE samples to WT EXE samples. Statistical analysis for DNA methylation was performed by using one-way ANOVA with Dunnett multiple comparison test. Statistical analysis values for imprinted gene expression were determined by DESeq2 for RNA-seq analysis of imprinted genes. The symbols represent the following values in statistical analysis on the figure: ∗, *p* < 0.05; ∗∗, *p* < 0.01; ∗∗∗, *p* < 0.001. ns, not statistically significant, with a *p*-value more than 0.1.

**Figure 4:**
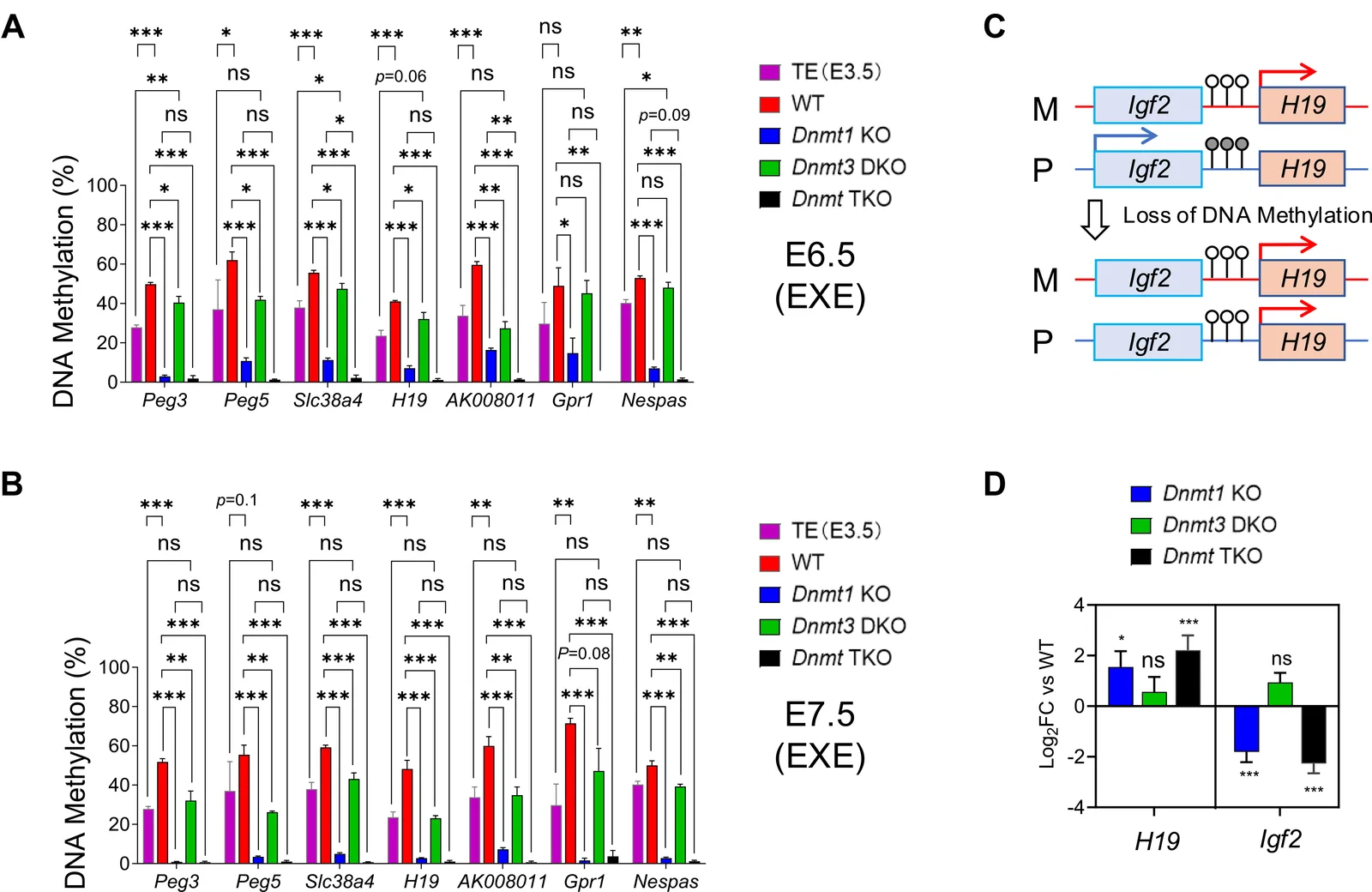
**DNMT3A and DNMT3B caused increased DNA methylation in seven imprinted regions in the extraembryonic ectoderm of the post-implantation embryos.** DNA methylation in the imprinting control region (ICR) of 24 known imprinted regions was examined for the genomic DNA samples derived from the trophectoderm (TE) of the wild-type (WT) E3.5 embryos, the extraembryonic ectoderm (EXE) of WT and *Dnmt* mutant E6.5 and E7.5 embryos. DNA methylation in 7 ICRs was statistically significantly increased in the EXE of WT E6.5-E7.5 embryos in compared to TE of WT E3.5 embryos. These ICRs include the ICRs of *Peg3, Peg5, Slc38a4, AK008011, Gpr1, Nespas,* and *H19* of *Igf2-H19* imprinted regions. *AK008011* ICR has an extended region identified in our previous study (6). Expression of the corresponding imprinted genes was also analyzed for these imprinted regions in the extraembryonic ectoderm (EXE) of WT and *Dnmt* mutant E6.5 and E7.5 embryos. *A*, DNA methylation was analyzed for 7 ICRs in the WT TE, the EXE of WT, *Dnmt1* mutant (*Dnmt1* KO), *Dnmt3* double mutant (*Dnmt3* DKO) and *Dnmt* triple mutant (*Dnmt* TKO) E6.5 embryos. *B*, DNA methylation was analyzed for 7 ICRs in the WT TE, the EXE of WT, *Dnmt1* mutant (*Dnmt1* KO), *Dnmt3* double mutant (*Dnmt3* DKO) and *Dnmt* triple mutant (*Dnmt* TKO) E7.5 embryos. *C*, the diagrams are used to illustrate expression of the *H19* and *Igf2* imprinted genes upon loss of DNA methylation at the *H19* ICR in the *Igf2-H19* imprinted region according to the allelic expression switch model. Filled lollipops, methylated CpG sites in the ICR. Unfilled lollipops, unmethylated CpG sites in the ICR. *D,* relative expression levels of the *H19* and *Igf2* imprinted genes were determined by DESeq2 with default settings comparing gene expression levels in the EXE samples of *Dnmt1* KO, *Dnmt3* DKO or *Dnmt* TKO mutant E7.5 embryos to the EXE samples of WT E7.5 embryos. Vertical axis, log_2_ fold change (log_2_FC) obtained from DESeq2 for relative expression levels of *H19* and *Igf2* comparing *Dnmt* mutant EXE samples to WT EXE samples. Statistical analysis for DNA methylation was performed by using one-way ANOVA with Dunnett multiple comparison test. Statistical analysis values for imprinted gene expression were determined by DESeq2 for RNA-seq analysis of imprinted genes. The symbols represent the following values in statistical analysis on the figure: ∗, *p* < 0.05; ∗∗, *p* < 0.01; ∗∗∗, *p* < 0.001. ns, not statistically significant, with a *p*-value more than 0.1.

**Figure 5:**
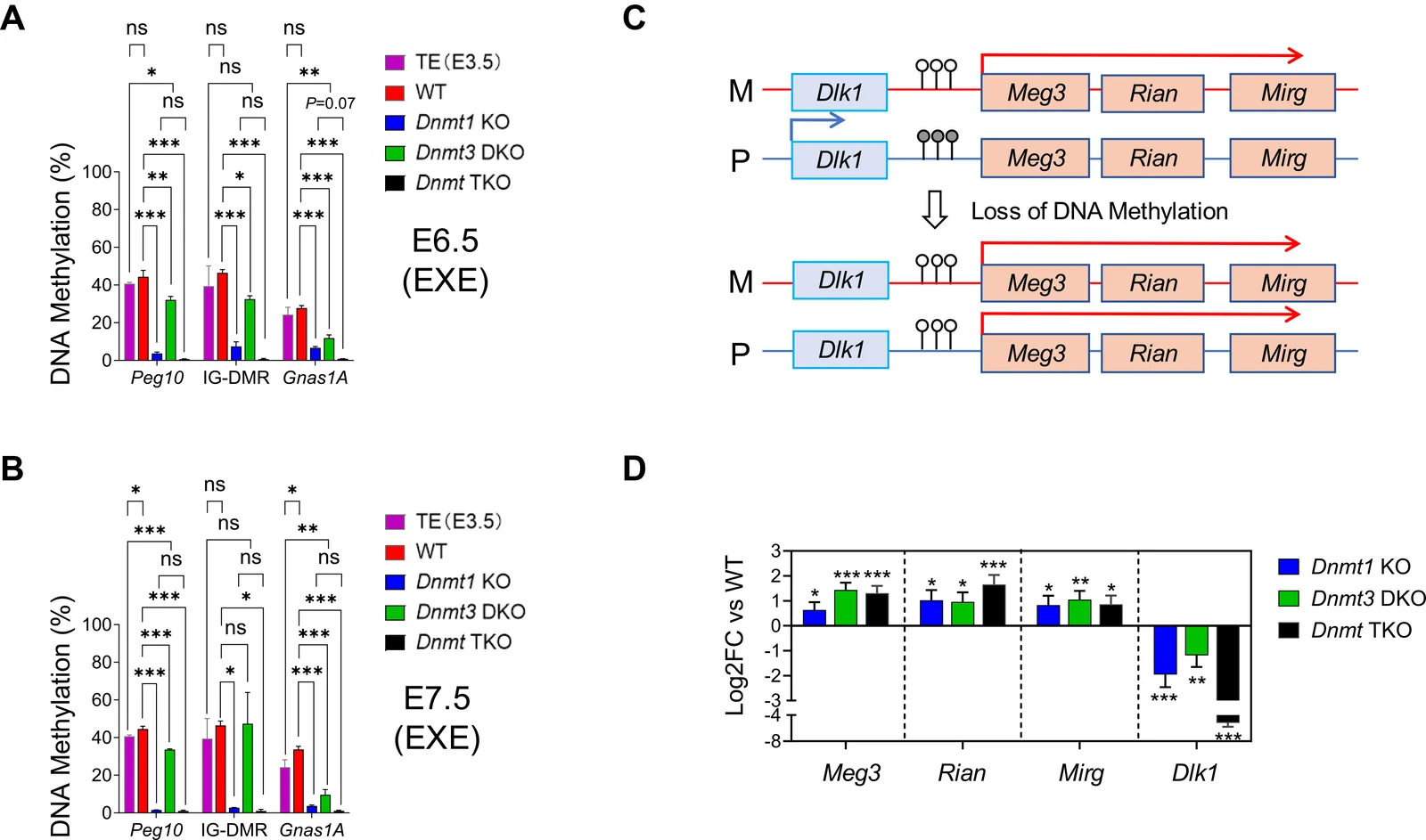
**DNMT3 proteins maintained pre-existing germline-derived DNA methylation in three imprinted regions in the extraembryonic ectoderm of the post-implantation embryos.** DNA methylation in the imprinting control region (ICR) of 24 known imprinted regions was examined for the genomic DNA samples derived from the trophectoderm (TE) of the wild-type (WT) E3.5 embryos, the extraembryonic ectoderm (EXE) of WT and *Dnmt* mutant E6.5 and E7.5 embryos. DNA methylation in 3 ICRs was statistically significantly lost in the EXE of *Dnmt3* DKO mutant E6.5-E7.5 embryos compared with the EXE of WT E6.5-E7.5 embryos. These ICRs include the ICRs of *Peg10, Gnas1A* and IG-DMR of the *Dlk1-Meg3* imprinted regions. Expression of the corresponding imprinted genes was also analyzed for these imprinted regions in the extraembryonic ectoderm (EXE) of WT and *Dnmt* mutant E6.5 and E7.5 embryos. *A*, DNA methylation was analyzed for 3 ICRs in the WT TE, the EXE of WT, *Dnmt1* mutant (*Dnmt1* KO), *Dnmt3* double mutant (*Dnmt3* DKO) and *Dnmt* triple mutant (*Dnmt* TKO) E6.5 embryos. *B*, DNA methylation was analyzed for 3 ICRs in the WT TE, the EXE of WT, *Dnmt1* mutant (*Dnmt1* KO), *Dnmt3* double mutant (*Dnmt3* DKO) and *Dnmt* triple mutant (*Dnmt* TKO) E7.5 embryos. *C*, the shown diagrams illustrate expression of the *Dlk1, Meg3, Rian* and *Mirg* imprinted genes upon loss of DNA methylation at the IG-DMR of the *Dlk1-Meg3* imprinted region according to the allelic expression switch model. Filled lollipops, methylated CpG sites in the ICR. Unfilled lollipops, unmethylated CpG sites in the ICR. *D*, relative expression levels of the *Dlk1, Meg3, Rian* and *Mirg* imprinted genes were determined by DESeq2 with default settings comparing gene expression levels in the EXE samples of *Dnmt1* KO, *Dnmt3* DKO or *Dnmt* TKO mutant E7.5 embryos to the EXE samples of WT E7.5 embryos. Vertical axis, log_2_ fold change (log_2_FC) obtained from DESeq2 for relative expression levels of *Dlk1, Meg3, Rian* and *Mirg* comparing *Dnmt* mutant EXE samples to WT EXE samples. Statistical analysis for DNA methylation was performed by using one-way ANOVA with Dunnett multiple comparison test. Statistical analysis values for imprinted gene expression were determined by DESeq2 for RNA-seq analysis of imprinted genes. The symbols represent the following values in statistical analysis on the figure: ∗, *p* < 0.05; ∗∗, *p* < 0.01; ∗∗∗, *p* < 0.001. ns, not statistically significant, with a *p*-value more than 0.1.

Interestingly, parent-of-origin-dependent monoallelic expression of the imprinted genes was affected when DNA methylation was lost at these ICRs in EXE of *Dnmt1* KO or *Dnmt* TKO mutant E6.5-E7.5 embryos. As shown for the imprinted genes in the *Mcts2* imprinted region, expression of the *H13* imprinted gene decreased in EXE of *Dnmt1* KO or *Dnmt* TKO mutant E7.5 embryos as it was silenced on both alleles, whereas expression of the *Mcts2* imprinted gene increased in EXE of *Dnmt1* KO or *Dnmt* TKO mutant E7.5 embryos as it was expressed from both maternal as well as paternal alleles (Fig. 3, *C* and *D*, and the figure legend for *H13* expression analysis) (Table S7). These results are consistent with the DNA methylation-dependent allelic expression switch model for the imprinted genes in the *Mcts2* imprinted region (Fig. 3*C*). Similar results were obtained for the *Snrpn*, *Ndn* and *Mkrn3* imprinted genes in the *Snrpn* imprinted region (Fig. 3, *E* and *F*) (Table S7). Expression of the *Snrpn* and *Mkrn3* imprinted genes increased in EXE of *Dnmt1* KO or *Dnmt* TKO mutant E7.5 embryos, whereas expression of the *Ndn* imprinted gene increased only in *Dnmt* TKO mutant E7.5 embryos (Fig. 3*F*, and the figure legend for possible explanation of *Ndn* expression). These results also fit the DNA methylation-dependent allelic expression switch model for the imprinted genes in the *Snrpn* imprinted region (Fig. 3*E*).

### DNA methylation was increased in seven imprinted regions in EXE after implantation

Similar to what we found in the epiblast of post-implantation embryos, DNA methylation significantly increased at a large subset of ICRs in EXE of WT E6.5-E7.5 embryos compared with TE of WT E3.5 embryos (Tables S4–S6) (Fig. 4, *A* and *B*) (6). About 20 to 40% of DNA methylation was obtained at the *Peg3*, *Peg5*, *Slc38a4*, *H19*, *AK008011*, *Gpr1*, and *Nespas* ICRs in TE of WT E3.5 embryos. DNA methylation increased to 50 to 60% at these ICRs in EXE of WT E6.5-E7.5 embryos except that it increased to over 70% at *Gpr1* ICR in EXE of WT E7.5 embryos (Fig. 4, *A* and *B*). DNA methylation was still significantly higher at *Peg3*, *Slc38a4*, *Nespas*, and *H19* ICRs in EXE of E6.5 *Dnmt3* DKO mutant embryos in comparison to TE of WT E3.5 embryos (Fig. 4*A*). But the increased DNA methylation was abolished at the *Peg5, Gpr1*, and *AK008011* ICRs in EXE of E6.5 *Dnmt3* DKO mutant embryos compared with TE of WT E3.5 embryos (Fig. 4*A*). This increase in DNA methylation was eliminated at these seven ICRs in EXE of E7.5 *Dnmt3* DKO mutant embryos in comparison to TE of WT E3.5 embryos (Fig. 4*B*). As expected, DNA methylation was almost completely missing at these seven ICRs in EXE of *Dnmt* TKO mutant E6.5-E7.5 embryos (Fig. 4, *A* and *B*). DNA methylation was largely lost at these seven ICRs in EXE of *Dnmt1* KO mutant E6.5-E7.5 embryos, although DNA methylation was still significantly higher at *Slc38a4* and *AK008011* ICRs, and maybe also at *Nespas* ICR, in EXE of *Dnmt1* KO mutant E6.5 embryos in comparison to *Dnmt* TKO mutant E6.5 embryos (Fig. 4, *A* and *B*). Interestingly, DNA methylation was significantly reduced at five ICRs (*Peg3, Peg5, Slc38a4, H19* and *AK008011*) in EXE of *Dnmt3* DKO mutant E6.5-E7.5 embryos in comparison to the WT E6.5-E7.5 embryos (Fig. 4, *A* and *B*). DNA methylation also appeared to be reduced at the *Gpr1* and *Nespas* ICRs in EXE of *Dnmt3* DKO mutant E7.5 embryos in comparison to the WT E7.5 embryos (Fig. 4, *A* and *B*).

Expression of the *H19* imprinted gene in the *Igf2-H19* imprinted region increased in EXE of *Dnmt1* KO or *Dnmt* TKO mutant E7.5 embryos in comparison to the WT E7.5 embryos (Fig. 4, *C* and *D*) (Table S7). In contrast, expression of the *Igf2* imprinted gene in the *Igf2-H19* imprinted region decreased in EXE of *Dnmt1* KO or *Dnmt* TKO mutant E7.5 embryos in comparison to the WT E7.5 embryos (Fig. 4, *C* and *D*). These results fit the predictions for *Igf2* and *H19* expression in EXE of *Dnmt1* KO or *Dnmt* TKO mutant E7.5 embryos according to the DNA methylation-dependent allelic expression switch model for the imprinted genes in the *Igf2-H19* imprinted region (Fig. 4*C*).

In summary, these results suggest that *de novo* DNA methylation mediated by DNMT3A and DNMT3B accounts for the increased DNA methylation at these seven ICRs in EXE of E6.5-E7.5 embryos, whereas DNMT1 is the major DNMT to maintain DNA methylation at these ICRs in EXE of E6.5-E7.5 embryos. DNMT3A and DNMT3B, together with DNMT1, appear to maintain DNA methylation in at least two of these ICRs, *Slc38a4* and *AK008011* ICRs, in EXE of E6.5 embryos. Loss of DNA methylation at the *H19* ICR results in allelic expression switch of *Igf2* and *H19* imprinted genes.

### DNMT3A and DNMT3B, together with DNMT1, maintained DNA methylation in three imprinted regions in EXE of mouse embryos

Similar to what we found in the epiblast of post-implantation embryos in a previous study, DNMT3A and DNMT3B appear to contribute to the maintenance of preexisting DNA methylation at a subset of ICRs in EXE of E6.5-E7.5 embryos (Fig. 5, *A* and *B*) (6). A similar level of DNA methylation was observed at three ICRs (*Peg10*, IG-DMR of *Dlk1-Meg3* imprinted region, and *Gnas1A*) in EXE of WT E6.5 embryos compared with TE of WT E3.5 embryos (Fig. 5*A*). DNA methylation was also similar at the IG-DMR in EXE of WT E7.5 embryos compared with TE of WT E3.5 embryos, although it was slightly but significantly increased at *Peg10* and *Gnas1A* ICRs in EXE of WT E7.5 embryos compared with TE of WT E3.5 embryos (Fig. 5*B*). Interestingly, DNA methylation was significantly reduced at *Peg10* and *Gnas1A* ICRs in EXE of *Dnmt3* DKO mutant E6.5-E7.5 embryos in comparison to the WT E6.5-E7.5 embryos (Fig. 5, *A* and *B*). DNA methylation was also significantly reduced at the IG-DMR in EXE of *Dnmt3* DKO mutant E6.5 embryos in comparison to the WT E6.5 embryos (Fig. 5*A*). However, DNA methylation was not significantly reduced at IG-DMR in EXE of *Dnmt3* DKO mutant E7.5 embryos in comparison to the WT E7.5 embryos, which may be due to relatively high variability in DNA methylation at IG-DMR in the samples derived from *Dnmt3* DKO mutant E7.5 embryos (Fig. 5*B*).

Interestingly, expression of the imprinted genes in the *Dlk1-Dio3* imprinted region was affected by loss of DNA methylation at the IG-DMR in *Dnmt1* KO, *Dnmt3* DKO, and *Dnmt* TKO mutant E7.5 embryos (Fig. 5, *C* and *D*) (Table S7). *Dlk1* expression decreased, whereas expression of *Meg3*, *Rian*, and *Mirg* increased in *Dnmt1* KO, *Dnmt3* DKO, and *Dnmt* TKO mutant E7.5 embryos (Fig. 5, *C* and *D*). Expression of these imprinted genes is consistent with the DNA methylation-dependent allelic expression switch model (Fig. 5*C*).

In summary, DNMT1 is the major DNMT for maintaining DNA methylation of all ICRs in EXE of E6.5-E7.5 embryos. However, DNMT3A and DNMT3B also play a role in maintaining DNA methylation at some ICRs in EXE of E6.5-E7.5 embryos. In addition, a large subset of ICRs display increased DNA methylation in EXE after implantation that results from *de novo* DNA methylation mediated by DNMT3A and DNMT3B.

### DNA methylation in the somatic differentially methylated regions (sDMRs) of some imprinted regions

DNA methylation in the secondary differentially methylated regions (DMRs) located in the imprinted regions is established in the somatic cells after fertilization (18, 28). Therefore, these secondary DMRs are also called somatic DMRs (sDMRs) that had been identified in some previous studies, with many analyzed in our current study (Table S8) (8, 38, 39, 40, 41, 42, 43, 44). First, we examined DNA methylation in some sDMRs that are located in the imprinted regions containing the ICRs with seemingly stable DNA methylation after implantation (Figs. 3 and S6) (Table S9). Compared with TE of WT E3.5 embryos, increased DNA methylation was observed at the *Igf2r* TSS DMR in the *Igf2r* imprinted region, *Cdkn1c*, *Slc22a18* and *Kcnq1* Intergenic 2 DMRs in the *Kcnq1ot1* imprinted region, and *Magel2* and *Ndn* DMRs in the *Snrpn* imprinted region in EXE of WT E6.5 or E7.5 embryos except for statistically insignificant increase of DNA methylation at *Ndn* DMR in EXE of WT E6.5 embryos, whereas DNA methylation decreased significantly at *Mkrn3* DMR in the *Snrpn* imprinted region in EXE of WT E6.5 or E7.5 embryos (Fig. S6). DNA methylation was not much different at *Peg12* and *Kcnq1* Intergenic 1 DMRs in EXE of WT E6.5 or E7.5 embryos in comparison to TE of WT E3.5 embryos (Fig. S6). Increased DNA methylation was eliminated at the sDMRs in EXE of *Dnmt3* DKO mutant E6.5 or E7.5 embryos, indicating the requirement of two DNMT3 proteins in *de novo* DNA methylation at these sDMRs after implantation (Fig. S6). Interestingly, DNA methylation was significantly reduced at the *Mkrn3* DMR in EXE of *Dnmt3* DKO mutant E6.5 or E7.5 embryos compared with TE of WT E3.5 embryos or EXE of the corresponding WT E7.5 embryos, suggesting that two DNMT3 proteins maintain the pre-existing DNA methylation at this sDMR (Fig. S6). This may be also the case for *Kcnq1* Intergenic 1 DMR as DNA methylation was significantly reduced in EXE of *Dnmt3* DKO mutant E6.5 embryos compared with EXE of WT E6.5 embryos, and close to be significantly reduced in EXE of *Dnmt3* DKO mutant E7.5 embryos in comparison to TE of WT E3.5 embryos (Fig. S6). In addition, DNA methylation was significantly higher at the *Igf2r* TSS DMR in *Dnmt1* KO mutant E6.5-E7.5 embryos in comparison to *Dnmt* TKO mutant E6.5-E7.5 embryos, indicating the functions of two DNMT3 proteins in maintaining newly acquired DNA methylation at this sDMR (Fig. S6).

Next, we examined DNA methylation at some sDMRs located in the imprinted regions with increased DNA methylation at the ICRs in EXE after implantation (Figs. 4 and S7) (Table S8 and S9). Compared with TE of WT E3.5 embryos, increased DNA methylation was also observed at most sDMRs in EXE of WT E6.5-E7.5 embryos that include the *Zim2* and *Zim3* DMRs in the *Peg3* imprinted region, *Zdbf2* DMR in the *Gpr1* imprinted region, *Nesp* DMR in the *Nespas* imprinted region, and *Igf2* DMR1 and *Igf2* DMR2 in the *Igf2-H19* imprinted region (Fig. S7) (Table S8 and S9). This gain of DNA methylation was abolished at these sDMRs in EXE of *Dnmt3* DKO mutant E6.5 or E7.5 embryos (Fig. S7, *A* and *B*). DNA methylation was still significantly higher at the *Igf2* DMR2 and *Nesp* DMRs in EXE of *Dnmt1* KO mutant E6.5-E7.5 embryos, and at the *Zim2* and *Zim3* DMRs in EXE of *Dnmt1* KO mutant E6.5 embryos in comparison to *Dnmt* TKO mutant E6.5-E7.5 embryos (Fig. S7, *A* and *B*). No increased DNA methylation was observed at the *Igf2* DMR0 in EXE of WT E6.5-E7.5 embryos in comparison to TE of WT E3.5 embryos, and DNA methylation was significantly reduced at the *Igf2* DMR0 in EXE of *Dnmt3* DKO mutant E6.5 embryos compared with TE of WT E3.5 embryos (Fig. S7, *C* and *D*). These results suggest that two DNMT3 proteins maintain both newly acquired and pre-existing DNA methylation at these sDMRs in EXE of post-implantation embryos, besides their functions in *de novo* DNA methylation at these sDMRs after implantation.

We also analyzed three sDMRs in the *Dlk1-Meg3* imprinted region (Fig. S8) (Tables S8 and S9). Increased DNA methylation was observed at *Meg3* and *Dlk1* DMRs in EXE of WT E6.5-E7.5 embryos compared with TE of WT E3.5 embryos (Fig. S8, *A* and *B*). This gain of DNA methylation was eliminated in these sDMRs in EXE of *Dnmt3* DKO mutant E6.5-E7.5 embryos in comparison to TE of WT E3.5 embryos (Fig. S8, *A* and *B*). No increased DNA methylation was observed at the *Rian* DMR in EXE of WT E6.5-E7.5 embryos in comparison to TE of WT E3.5 embryos, and DNA methylation was significantly reduced at the *Rian* DMR in EXE of *Dnmt3* DKO mutant E6.5 or E7.5 embryos compared with TE of WT E3.5 embryos or EXE of the corresponding WT E7.5 embryos (Fig. S8, *C* and *D*).

In summary, DNA methylation increases at many somatic DMRs in EXE after implantation. This gain-of-DNA methylation is mediated by DNMT3A and DNMT3B. Together with DNMT1, they also maintain both newly acquired and preexisting DNA methylation at somatic DMRs in EXE after implantation.

## Discussion

DNMT3A and DNMT3B appear to be required for increased DNA methylation in the EXE of post-implantation embryos through their *de novo* DNA methyltransferase activities. This is similar to what we had found before about their roles in *de novo* DNA methylation in the epiblast of post-implantation embryos in a previously published study (Fig. 6) (6). Interestingly, there was increased DNA methylation at the ICRs of a large subset of imprinted regions mediated by *de novo* DNA methyltransferase activities of DNMT3A and DNMT3B in the EXE of post-implantation embryos, which is also similar to what was observed in our previous study about increased DNA methylation in a subset of imprinted regions in the epiblast of post-implantation embryos (Fig. 6) (6). Taken together, these results suggest that DNA methylation at the ICRs may be more dynamically regulated than previously thought in the post-implantation embryos. It is unclear why only a small subset of imprinted regions seems to be more susceptible to *de novo* DNA methylation mediated by DNMT3A and DNMT3B, and different ICRs experienced increased DNA methylation in the epiblast *versus* EXE after implantation.

**Figure 6:**
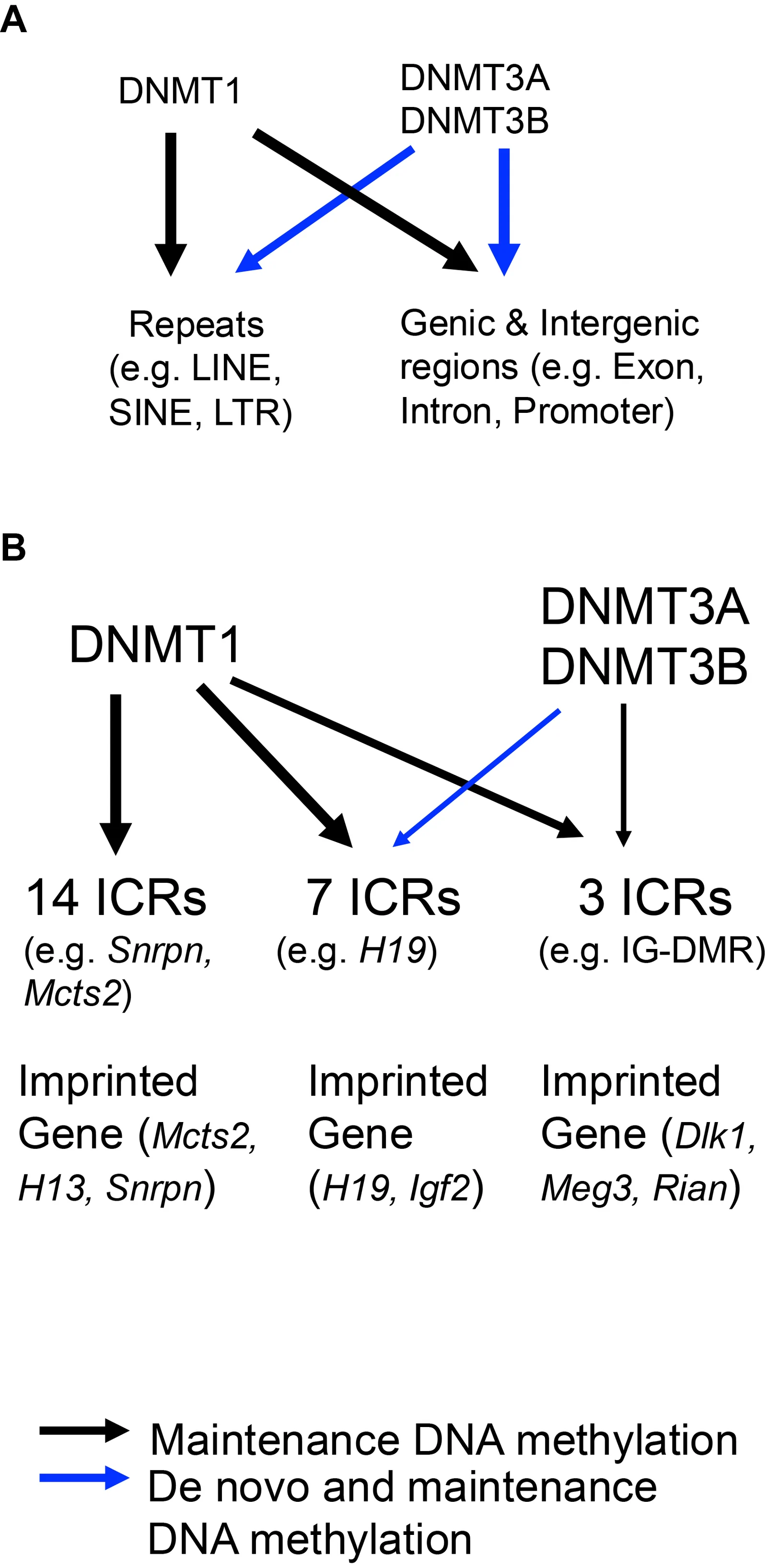
**The schematic models show the functions of three DNMT proteins in DNA methylation and imprinted gene expression in the extraembryonic ectoderm of the post-implantation embryos.** Two DNMT3 proteins, together with DNMT1, may maintain DNA methylation in the repeats, genic and intergenic regions in the extraembryonic ectoderm (EXE) of post-implantation embryos, as indicated by the *black arrows* in the figures. DNMT3A and DNMT3B may function in both *de novo* DNA methylation and maintenance DNA methylation, which is marked by the *blue arrows* in the figures. The line thickness of the *arrows* is in proportion to their estimated roles in DNA methylation according to this study. *A*, the functions of three DNMT proteins (DNMT1, DNMT3A and DNMT3B) in DNA methylation in the repeats, genic and intergenic regions in EXE of post-implantation embryos. DNMT1 maintains DNA methylation in the repeats (*e.g.* LINE, SINE and LTR), genic and intergenic regions (*e.g.* Exon, Intron, Promoter). Interestingly, DNMT3A and DNMT3B function in the maintenance DNA methylation as well as *de novo* DNA methylation at these genomic regions in EXE after implantation. *B*, DNMT1, DNMT3A and DNMT3B regulate DNA methylation at the ICRs in the imprinted regions and expression of the corresponding imprinted genes in EXE of the post-implantation embryos. Loss of DNA methylation at the ICR causes allelic expression switch of the corresponding imprinted genes regulated by this ICR. There are three groups of ICRs based on the functions of three DNMT proteins in ICR DNA methylation. Group 1, 14 ICRs. DNMT1 maintains pre-existing DNA methylation at 14 ICRs, including *Snrpn* and *Mcts2*. It also regulates mono-allelic expression of the imprinted genes such as *Mcts2, H13* and *Snrpn* in EXE of E7.5 embryos. Group 2, 7 ICRs (*Peg3*, *Peg5*, *Slc38a4*, *H19*, *AK008011, Gpr1, and Nespas* ICR). DNMT3A and DNMT3B function in *de novo* DNA methylation at seven other ICRs, such as *H19* ICR. Together with DNMT1, they maintain DNA methylation at these seven ICRs in the EXE of post-implantation embryos. Interestingly, DNMT3A and DNMT3B also contribute to maintaining DNA methylation in at least two of these ICRs. *H19* is bi-allelically expressed, whereas *Igf2* is turned off on both paternal and maternal alleles upon loss of DNA methylation at the *H19* ICR in EXE of *Dnmt1* KO or *Dnmt* TKO E7.5 embryos. Group 3, 3 ICRs (*Peg10, Gnas1A* and IG-DMR of the *Dlk1-Meg3* imprinted region). Although DNMT1 is the major DNMT for maintaining preexisting DNA methylation at three other ICRs that include the IG-DMR of the *Dlk1-Meg3* imprinted region, DNMT3A and DNMT3B contribute to the maintenance of DNA methylation at these ICRs in EXE of post-implantation embryos. It appears that *Dlk1* is turned off on both paternal and maternal alleles, whereas *Meg3, Rian, and Mirg* are bi-allelically expressed upon loss of DNA methylation at the IG-DMR in EXE of *Dnmt1* KO, *Dnmt3* DKO or *Dnmt* TKO E7.5 embryos.

Similar to what was observed in the epiblast of post-implantation embryos as well as in ES cells, DNMT3A and DNMT3B, together with DNMT1, maintain DNA methylation across the genome including repeats, genic and intergenic regions (Fig. 6) (6). Furthermore, DNMT3A and DNMT3B contribute to the maintenance of preexisting DNA methylation at the promoters and exons as well as Simple Repeat and Low Complexity regions in EXE after implantation, which was not observed in these regions in epiblast of post-implantation embryos in our previous study (Fig. 2) (6). They also play important roles in maintaining pre-existing DNA methylation in the *Gata4* gene in EXE after implantation. Interestingly, DNMT3A and DNMT3B are required for maintaining pre-existing DNA methylation at CGIs and newly acquired DNA methylation in the *Nanog* gene in both EXE and epiblast after implantation (Figs. S3 and S4) (6). These findings underscore the critical functions of DNMT3A and DNMT3B in both *de novo* and maintenance DNA methylation across the genome after implantation.

Although DNMT1 plays primary roles in maintaining DNA methylation at most ICRs, DNMT3A and DNMT3B make significant contributions to the maintenance of DNA methylation at a subset of ICRs in EXE of post-implantation embryos (Fig. 6). These results are also similar to what were observed in the epiblast of post-implantation embryos (6). However, it is not clear why different ICRs require DNMT3A and DNMT3B for the maintenance of DNA methylation in the epiblast *versus* EXE after implantation.

It is known that the ICRs as well as germline-derived differential DNA methylation at the ICRs are required for maintaining parent-of-origin-dependent mono-allelic expression of imprinted genes (45, 46). In addition, loss of the key regulator ZFP57 causes loss of DNA methylation at most known ICRs, which results in an allelic expression switch of the corresponding imprinted genes in mouse embryos (30). Interestingly, we also observed allelic expression switch of some exemplified imprinted genes upon loss of DNA methylation at the ICRs of these imprinted regions in the EXE of post-implantation embryos (Figure 3, Figure 4, Figure 5, Figure 6). These results provide further evidence for the DNA methylation-dependent allelic expression switch model for imprinted genes. Consistent with this model, DNMT3A and DNMT3B, together with DNMT1, maintain mono-allelic expression of imprinted genes at the *Dlk1-Meg3* imprinted region in the EXE after implantation, and allelic expression switch appears to occur at the imprinted genes at that imprinted region without DNMT1 or two DNMT3 proteins, or in the absence of three DNMT proteins.

It is possible that DNMT1 may still be sufficient to maintain part of newly acquired DNA methylation at the *Peg3*, *Slc38a4*, *Nespas*, and *H19* ICRs in EXE of E6.5 embryos, as DNA methylation was significantly higher in EXE of *Dnmt3* DKO mutant E6.5 embryos compared with TE of WT E3.5 embryos. However, DNMT1 is insufficient to maintain newly acquired DNA methylation in EXE of E7.5 embryos as the increased DNA methylation was essentially lost at these ICRs too in EXE of *Dnmt3* DKO mutant E7.5 embryos. Another possibility is that DNMT1, together with two DNMT3 proteins, caused increased DNA methylation at these ICRs in EXE of E6.5 embryos, although DNMT1 did not show any *de novo* DNA methylation activity at these ICRs in EXE of E7.5 embryos. We also think that increased DNA methylation present at these ICRs in EXE of *Dnmt3* DKO mutant E6.5 embryos may be due to maintenance DNA methylation mediated by residual DNMT3A and/or DNMT3B proteins that was inherited either from maternal deposition or from lasting gene product that persisted after CRISPR experiments in early embryos. Then the residual product was depleted at E7.5, and newly acquired DNA methylation was completely lost at these ICRs in EXE of *Dnmt3* DKO mutant E7.5 embryos. It will be interesting to distinguish these possibilities in the future.

Germline-derived differential DNA methylation is usually located at the ICRs on either the paternal chromosome or the maternal chromosome (20). However, DNA methylation seemed to be much higher than 50% in a small subset of ICRs in EXE of WT E6.5 or E7.5 embryos (Figs. 3 and 4) (Tables S5 and S6). This suggests that DNA methylation may be present at the ICRs on both parental chromosomes. It is reported that DNA methylation occurred at the *Gpr1* ICR on the paternal chromosome in E6.5-E7.5 embryos, in addition to its methylated maternal *Gpr1* ICR, with DNA methylation established during oogenesis (47, 48). In addition, DNA methylation was found to be present at the *Gpr1* ICR on the paternal chromosome in the visceral yolk sac of E9.5 embryos and in the placenta of E17.5 embryos that is partly derived from EXE (47). DNA methylation was shown to be acquired on the paternal chromosome at the *AK008011* ICR in some adult tissues such as liver, and at *Cdh15* ICR in ES cells and some adult brain tissues (49). We also found that DNA methylation was present at the *Cdh15* ICR on both paternal and maternal chromosomes in ES cells (8). Interestingly, DNA methylation increased at the *Gpr1* ICR and *AK008011* ICR in EXE after implantation according to our current study (Fig. 4). Therefore, it appears that *de novo* DNA methylation at the ICR at or after implantation may result in gain of DNA methylation at the originally unmethylated ICRs in EXE of post-implantation embryos.

Besides germline-derived differential DNA methylation at the ICRs, differential DNA methylation is acquired at some somatic DMRs (sDMRs) in the imprinted regions after fertilization. Increased DNA methylation was observed at most sDMRs in EXE after implantation, which was mediated by DNMT3A and DNMT3B (Figs. S6–S8). It is worth noting that gain of DNA methylation still occurred at some sDMRs in EXE of post-implantation embryos, although differential DNA methylation remained relatively stable at the ICRs of the corresponding imprinted regions (Figs. 3 and S6). Interestingly, two DNMT3 proteins, together with DNMT1, are required for maintaining both newly acquired and pre-existing DNA methylation at these sDMRs in EXE of post-implantation embryos, similar to their roles at the ICRs. It seems that dynamic changes in DNA methylation occur at at least some ICRs and most sDMRs in EXE upon implantation.

It is presently unclear why two DNMT3 proteins are only required for maintaining DNA methylation in a large subset of ICRs in EXE of post-implantation embryos, which partially overlap with those ICRs requiring maintenance functions of DNMT3 proteins in epiblast after implantation (Fig. 6*B*) (6). Increased DNA methylation was observed in some of these ICRs in EXE of post-implantation embryos which also share some ICRs with those experiencing increased DNA methylation in epiblast after implantation (Fig. 6*B*) (6). In the current study, we could not tell if there is any difference for the functions of DNMT3A and DNMT3B in *de novo* and maintenance DNA methylation in EXE after implantation. It is known that there are multiple different isoforms of DNMT3A and DNMT3B that show some differences in their activities and functions (50, 51, 52). It is also reported that SIN3A containing HDAC1 and HDAC2 interacts with DNMT3A2, which is required for establishment of DNA methylation at a subset of ICRs in oocytes (53). The activity of DNMT3B is shown to be regulated by modified histone H3 peptide (54). It will be interesting to find out if chromatin structures including histone modifications may play some roles in regulating DNA methylation mediated by DNMT3A and DNMT3B at these ICRs in EXE or epiblast of post-implantation embryos.

## Experimental procedures

### Whole-genome bisulfite sequencing (WGBS) analysis

#### Processing of WGBS data

The raw WGBS sequencing data were downloaded from the Gene Expression Omnibus (GEO) database according to two previously published studies (34, 35). Then they were processed using a standardized pipeline. Adapter trimming and quality filtering were performed using Trim Galore (v0.6.10) (Table 1).

**Table 1:** Key resources for DNA methylation and RNA-seq analyses

Deposited data		
WGBS data for the trophectoderm (TE) of E3.5 wild-type mouse embryos	Smith, Z.D., 2017	GEO: GSE84235
WGBS and RNA-seq data for the extraembryonic ectoderm (EXE) of E6.5 and E7.5 mouse embryos	Li, Q., 2023	GEO: GSE162903
Software and algorithms		
BitMapperBS (v1.0.2.1)	Cheng, H., 2018	https://github.com/chhylp123/BitMapperBS
BedTools (v2.31.0)	Quinlan and Hall, 2010	https://bedtools.readthedocs.io/en/latest/
Trim Galore (v0.6.10)	Babraham Institute	https://www.bioinformatics.babraham.ac.uk/projects/trim_galore/
Picard MarkDuplicates (v2.18.29)	Broad Institute	https://broadinstitute.github.io/picard/
MethylDackel (v0.3.0)		https://github.com/dpryan79/MethylDackel

The WGBS data for the TE samples of wild-type (WT) E3.5 embryos, with the GEO accession number GSE84235, were originally generated from conventional WGBS libraries in a previous study (35) (Table 1). The sequence reads of the WGBS data for the TE samples were first trimmed and filtered using Trim Galore (v0.6.10). Then the trimmed and filtered sequence reads were aligned in paired-end mode to the mouse reference genome (mm10) using BitMapperBS (v1.0.2.1) with the “--sensitive” option during the alignment process (Table 1).

The WGBS data for the extraembryonic ectoderm (EXE) samples of E6.5 and E7.5 embryos, with the GEO accession number GSE162903, were produced with a post-bisulfite adaptor tagging (PBAT) method for making sequencing libraries in a previously published study (34) (Table 1). A multi-step alignment strategy was applied to improve mapping efficiency for the WGBS sequence reads of EXE samples. Trimmed paired-end sequence reads were first aligned in paired-end mode using BitMapperBS with the “--sensitive” option, while unmapped sequence reads were still retained for further mapping. Unmapped sequence read pairs were then extracted from the resulting BAM files using samtools view (-f 4) and converted back to FASTQ format. These sequence reads were subsequently realigned in single-end mode to maximize read recovery. Due to the asymmetric structure of PBAT libraries, read-specific alignment parameters were applied, whereby read 1 (R1) was aligned using PBAT-specific settings (--pbat), while read 2 (R2) was aligned using standard bisulfite mapping parameters. The alignment files from the paired-end mapping and the single-end realignment steps were merged using samtools to generate the final BAM files for downstream analyses.

The duplicated sequence reads produced by PCR amplification were removed using Picard MarkDuplicates (v2.18.29) (Table 1). The CpG methylation calling was extracted using MethylDackel (v0.3.0) (Table 1). Only CpG sites covered by at least three sequence reads were retained for downstream analyses. DNA methylation level at individual CpG sites was calculated as the ratio of methylated reads to total reads covering the CpG site based on BedGraph output files.

### Genomic feature annotation and DNA methylation analysis

Genomic features were defined based on annotations from the UCSC Genome Browser (mm10). CpG islands (CGIs) and gene annotations (exon, intron, and intergenic regions) were obtained from UCSC CpG Island and RefSeq tracks, respectively. Promoter regions were defined as ±2 kb from transcription start sites. Repeat elements, including LINE, SINE, and LTR families, were obtained from RepeatMasker tracks. The imprinting control regions (ICRs) were defined according to our previously published paper (6) (Table S3).

Feature-level aggregation and intersection with genomic annotations were performed using BedTools (v2.31.0). DNA methylation levels across genomic features were calculated by averaging CpG methylation values within each genomic region.

### Statistical analysis

Statistical analyses were performed using one-way ANOVA followed by Dunnett’s multiple comparison test when the *Dnmt* mutant EXE samples were compared with wild-type EXE or TE samples. Similar statistical analyses were carried out for comparing *Dnmt1* KO or *Dnmt3* DKO mutant EXE samples with *Dnmt* TKO mutant EXE samples. Data are presented as mean ± SEM. Statistical significance is defined as follows: *p* < 0.05 (∗), *p* < 0.01 (∗∗), and *p* < 0.001 (∗∗∗). NS, not statistically significant, with a *p*-value more than 0.1.

### RNA-seq data processing and analysis

The original RNA-seq data for EXE of E7.5 embryos, with the GEO accession number GSE162903, were obtained from the GEO database according to the previously published study (34) (Table 1). Raw sequencing reads were processed using Trim Galore (v0.6.10) for adapter trimming and quality filtering. Filtered paired-end sequence reads were aligned to the mouse reference genome (UCSC mm39) and quantified using RSEM (v1.3.1), with STAR (v2.5.2b) employed as the underlying aligner (*via* rsem-calculate-expression --star --paired-end). The STAR genome index was generated using RefSeq gene annotations (NCBI RefSeq, release 2024-02-14) corresponding to the mm39 genome build, with the --sjdbOverhang parameter set to 100. The --estimate-rspd option was enabled to account for non-uniform read distribution.

Lowly expressed genes, with total counts < 10 across all samples, were filtered out and excluded from further analyses. Differential gene expression analysis was performed using DESeq2 with default settings, comparing gene expression levels in the EXE samples of *Dnmt* mutants. For data visualization, log_2_ fold change (log_2_FC) was obtained from DESeq2, and variability was represented as ± SEM of log_2_FC. Statistical significance was determined based on DESeq2-derived *p*-values and is indicated in the corresponding figures.

## Data availability

The WGBS data were downloaded from the GEO database with the accession number GSE84235 for the TE samples of E3.5 embryos and *GSE162903* for the EXE samples of E6.5 and E7.5 embryos. No raw WGBS data need to be transferred to the public databases if the results for this study are published later. For further information, please send the requests to the lead contact Dr Xiajun Li by email.

## Supporting information

Supporting information for this study includes Supplemental Figures, the legend for these Supplemental Figures and Supplemental Tables.

## Conflict of interest

The authors declare that they have no conflicts of interest with the contents of this article.
