# Supplementary material for: Mammalian DNA methyltransferases in DNA methylation and imprinted gene expression in extraembryonic ectoderm of post-implantation embryos
Source: J Biol Chem. 2026 Sep 11;302(10):113554. doi: 10.1016/j.jbc.2026.113554 (PMC13671026; doi:10.1016/j.jbc.2026.113554)
Supplement: Supplementary file 1 [file mmc1.pdf]

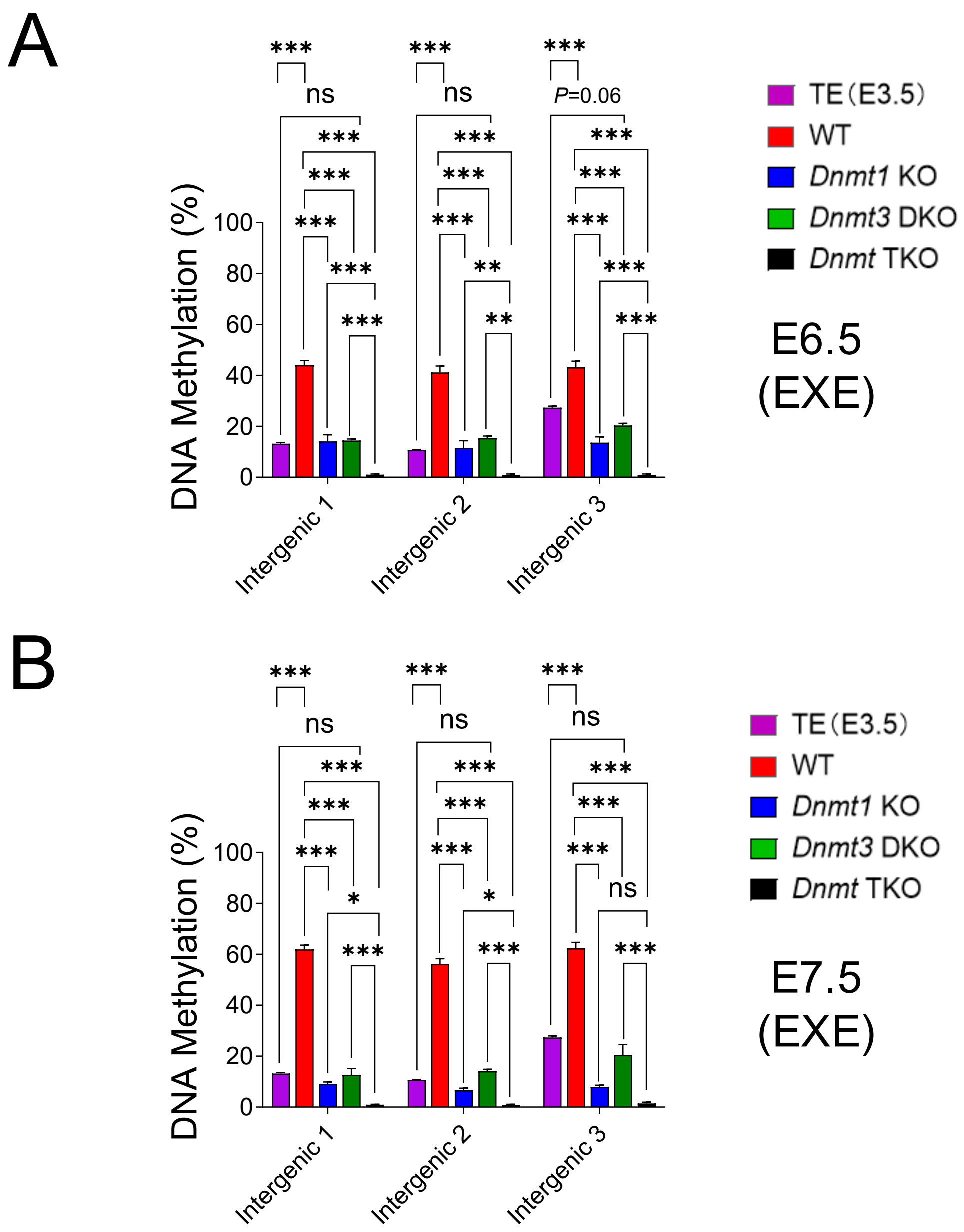
A
***
ns
***
***
***
***
***
DNA Methylation (%)
100
80
60
40
20
0
Intergenic 1
***
ns
***
***
***
**
**
Intergenic 2
***
P=0.06
***
***
***
***
***
Intergenic 3
TE（E3.5）
WT
Dnmt1 KO
Dnmt3 DKO
Dnmt TKO
E6.5
(EXE)
B
***
ns
***
***
***
*
***
DNA Methylation (%)
100
80
60
40
20
0
Intergenic 1
***
ns
***
***
***
*
***
Intergenic 2
***
ns
***
***
***
ns
***
Intergenic 3
TE（E3.5）
WT
Dnmt1 KO
Dnmt3 DKO
Dnmt TKO
E7.5
(EXE)

Fig. S1

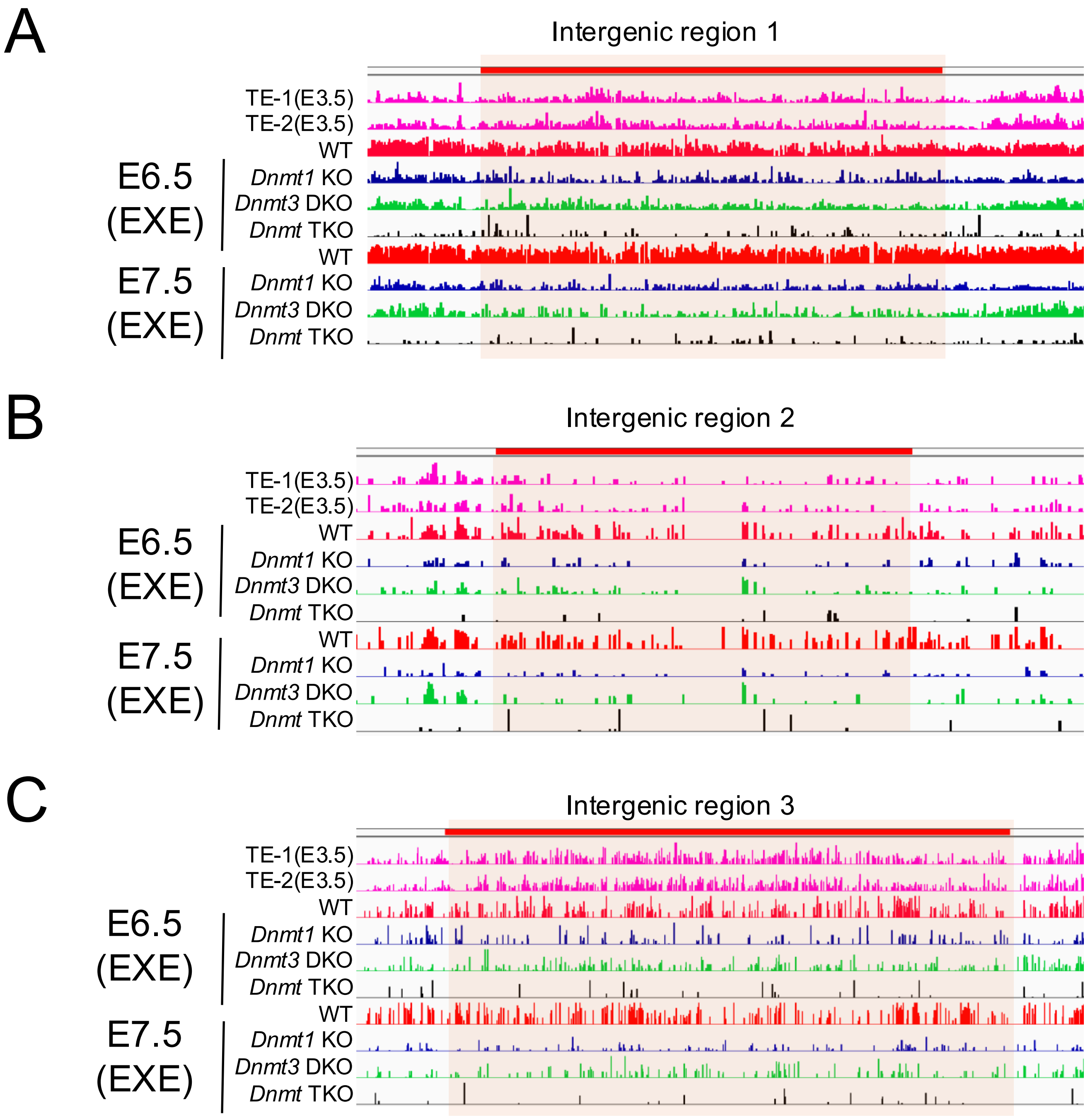
A
Intergenic region 1
TE-1(E3.5)
TE-2(E3.5)
WT
Dnmt1 KO
Dnmt3 DKO
Dnmt TKO
WT
Dnmt1 KO
Dnmt3 DKO
Dnmt TKO
E6.5 (EXE)
E7.5 (EXE)
B
Intergenic region 2
TE-1(E3.5)
TE-2(E3.5)
WT
Dnmt1 KO
Dnmt3 DKO
Dnmt TKO
WT
Dnmt1 KO
Dnmt3 DKO
Dnmt TKO
E6.5 (EXE)
E7.5 (EXE)
C
Intergenic region 3
TE-1(E3.5)
TE-2(E3.5)
WT
Dnmt1 KO
Dnmt3 DKO
Dnmt TKO
WT
Dnmt1 KO
Dnmt3 DKO
Dnmt TKO
E6.5 (EXE)
E7.5 (EXE)

Fig. S2

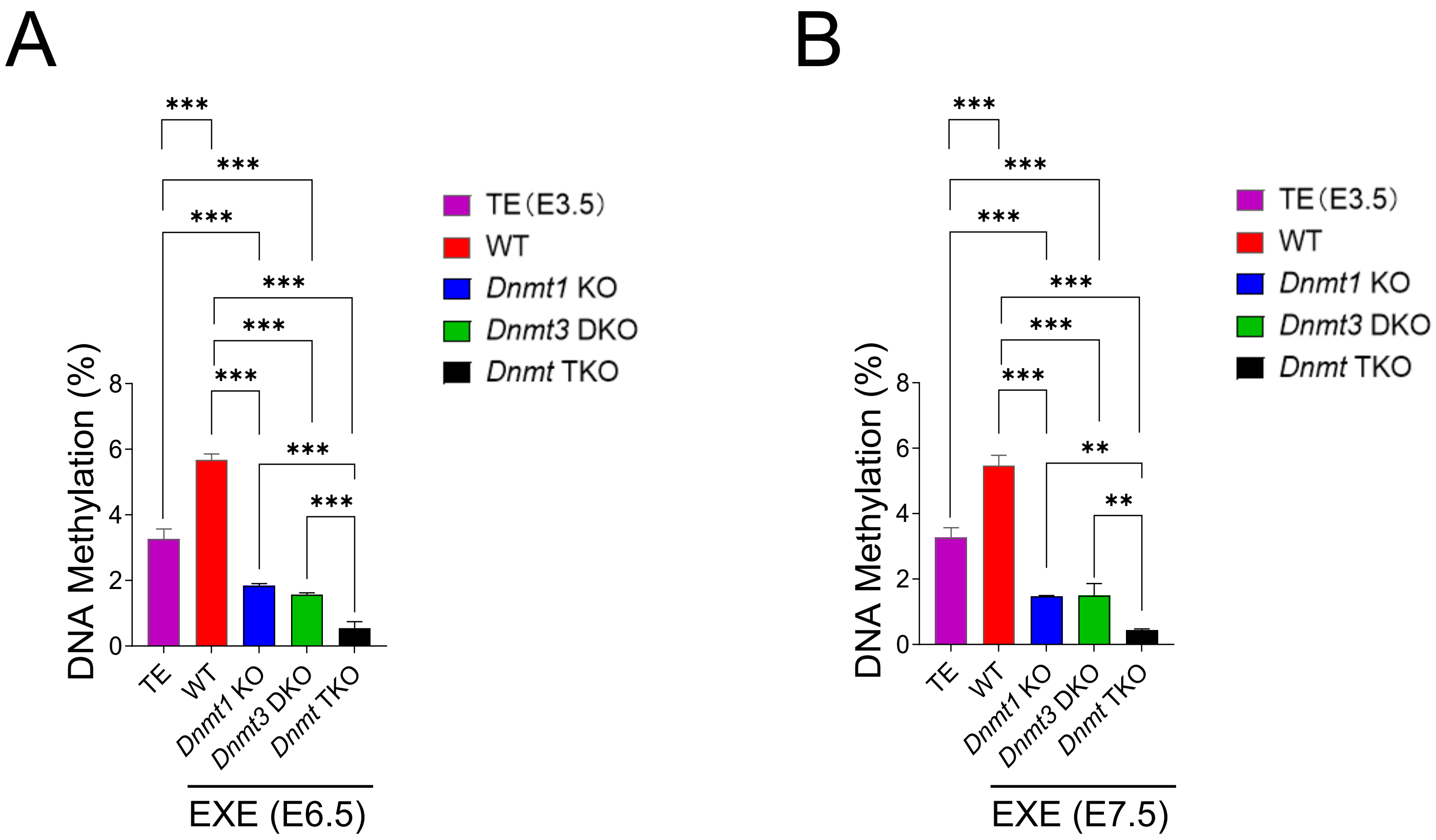

A
B
***
**
DNA Methylation (%)
0
2
4
6
8
TE
WT
Dnmt1 KO
Dnmt3 DKO
Dnmt TKO
EXE (E6.5)
EXE (E7.5)
TE（E3.5）

Fig. S3

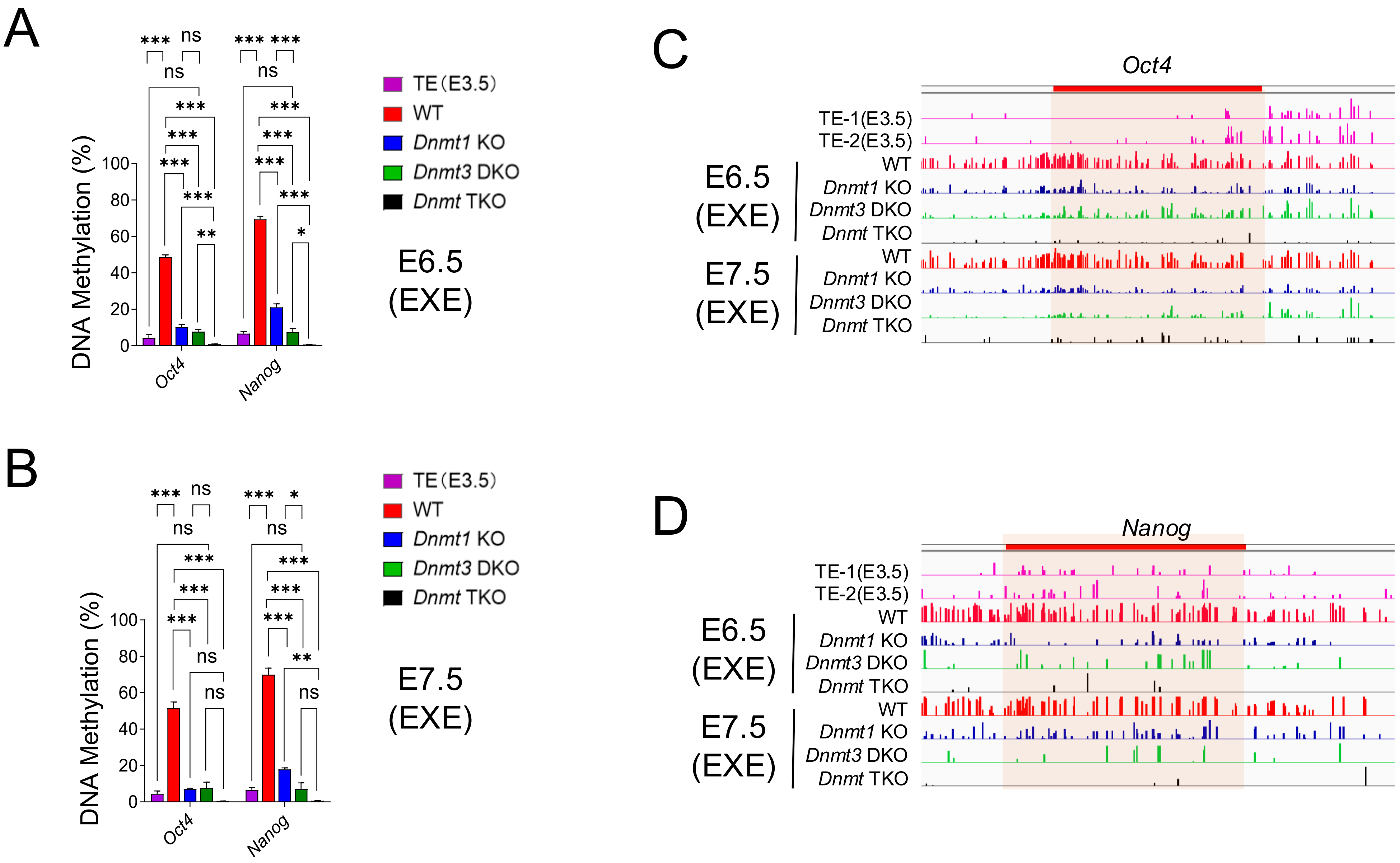
A
DNA Methylation (%)
0
20
40
60
80
100
Oct4
Nanog
***
ns
***
***
ns
ns
***
***
***
***
***
***
***
***
**
*
TE (E3.5)
WT
Dnmt1 KO
Dnmt3 DKO
Dnmt TKO
E6.5
(EXE)
B
DNA Methylation (%)
0
20
40
60
80
100
Oct4
Nanog
***
ns
***
*
ns
ns
***
***
***
***
***
***
ns
**
ns
ns
TE (E3.5)
WT
Dnmt1 KO
Dnmt3 DKO
Dnmt TKO
E7.5
(EXE)
C
Oct4
TE-1(E3.5)
TE-2(E3.5)
E6.5
(EXE)
WT
Dnmt1 KO
Dnmt3 DKO
Dnmt TKO
E7.5
(EXE)
WT
Dnmt1 KO
Dnmt3 DKO
Dnmt TKO
D
Nanog
TE-1(E3.5)
TE-2(E3.5)
E6.5
(EXE)
WT
Dnmt1 KO
Dnmt3 DKO
Dnmt TKO
E7.5
(EXE)
WT
Dnmt1 KO
Dnmt3 DKO
Dnmt TKO

Fig. S4

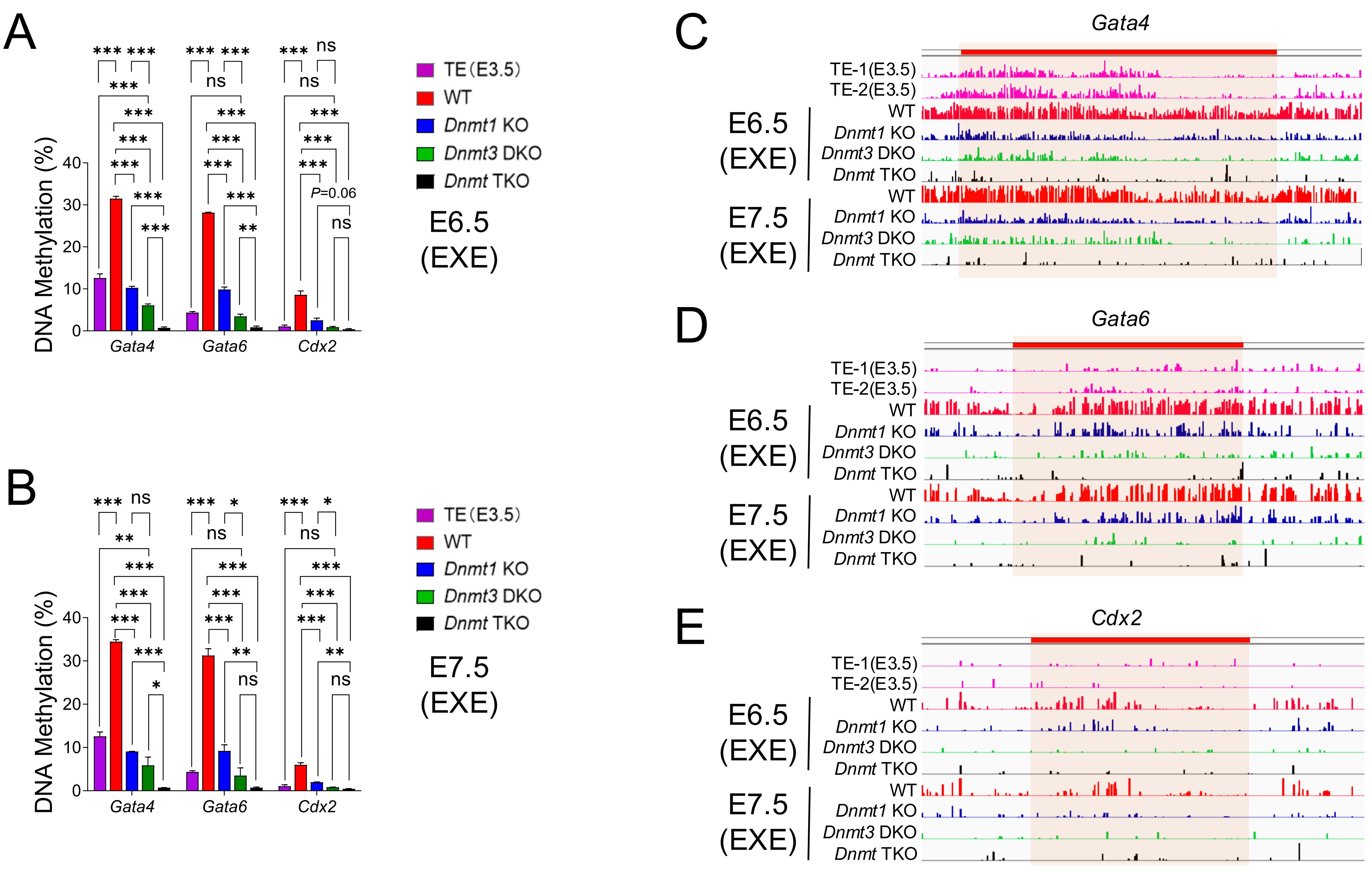

A
B
C
D
E
DNA Methylation (%)
0
10
20
30
40
Gata4
Gata6
Cdx2
***
**
*
ns
P=0.06
TE(E3.5)
WT
Dnmt1 KO
Dnmt3 DKO
Dnmt TKO
E6.5
(EXE)
E7.5
(EXE)
TE-1(E3.5)
TE-2(E3.5)

Fig. S5

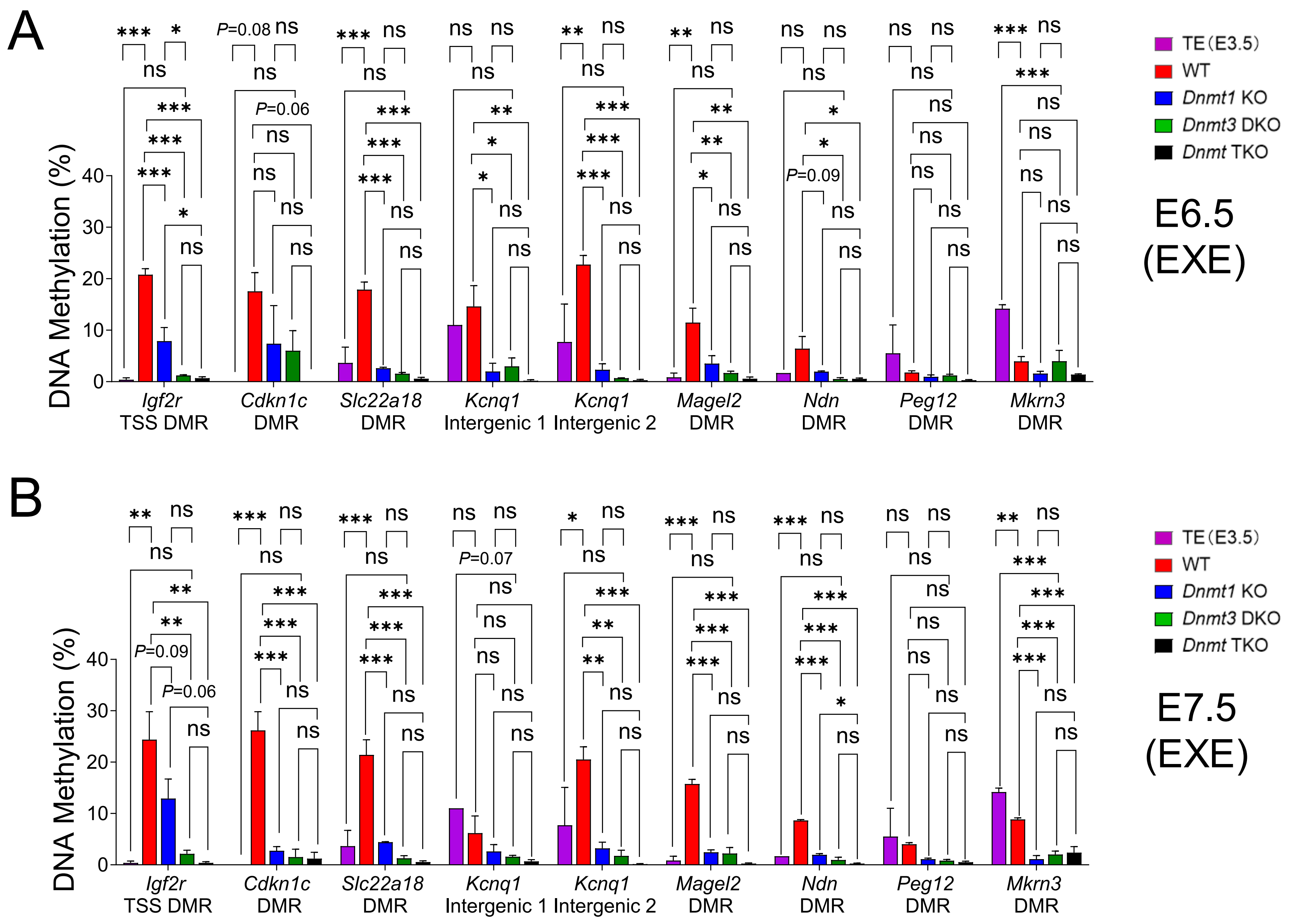
A
B
DNA Methylation (%)
0
10
20
30
40
Igf2r TSS DMR
Cdkn1c DMR
Slc22a18 DMR
Kcnq1 Intergenic 1
Kcnq1 Intergenic 2
Magel2 DMR
Ndn DMR
Peg12 DMR
Mkrn3 DMR
P=0.08
P=0.06
P=0.09
P=0.09
P=0.06
P=0.07
TE(E3.5)
WT
Dnmt1 KO
Dnmt3 DKO
Dnmt TKO
E6.5 (EXE)
E7.5 (EXE)

Fig. S6

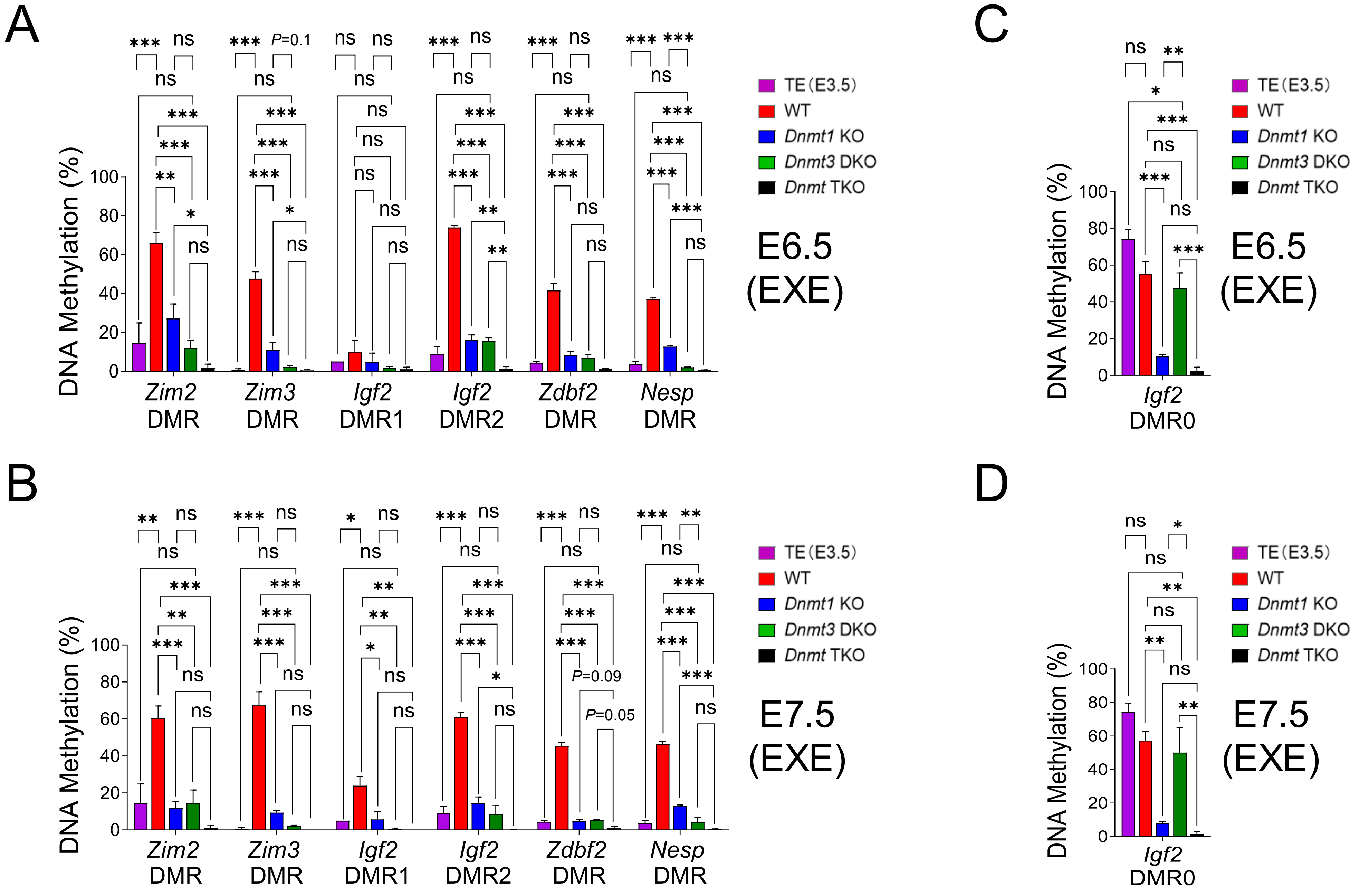
A
B
C
D
DNA Methylation (%)
Zim2 DMR
Zim3 DMR
Igf2 DMR1
Igf2 DMR2
Zdbf2 DMR
Nesp DMR
Igf2 DMR0
TE (E3.5)
WT
Dnmt1 KO
Dnmt3 DKO
Dnmt TKO
E6.5 (EXE)
E7.5 (EXE)
P=0.1
P=0.09
P=0.05

Fig. S7

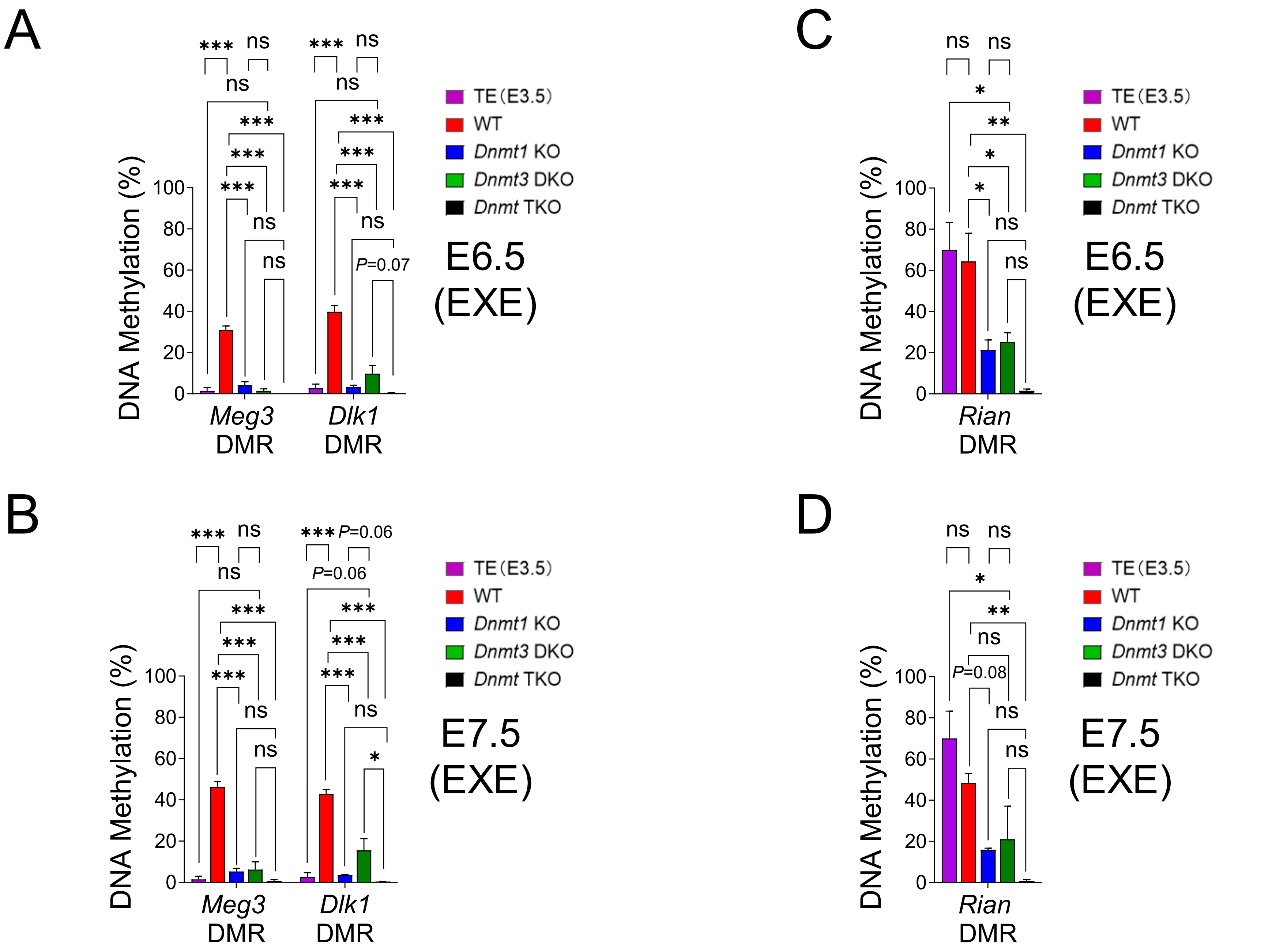
A
B
C
D
DNA Methylation (%)
0
20
40
60
80
100
Meg3
DMR
Dlk1
DMR
Rian
DMR
***
ns
*
**
P=0.07
P=0.06
P=0.08
TE (E3.5)
WT
Dnmt1 KO
Dnmt3 DKO
Dnmt TKO
E6.5
(EXE)
E7.5
(EXE)

Fig. S8
