# Supplementary material for: Mammalian DNA methyltransferases in DNA methylation and imprinted gene expression in extraembryonic ectoderm of post-implantation embryos
Source: J Biol Chem. 2026 Sep 11;302(10):113554. doi: 10.1016/j.jbc.2026.113554 (PMC13671026; doi:10.1016/j.jbc.2026.113554)
Supplement: Supplementary file 2 [file mmc2.pdf]

Supplemental Data

Mammalian DNA methyltransferases in DNA methylation and imprinted gene expression in extraembryonic ectoderm of post-implantation embryos

Ting Lu[1,#], Yilin Li[1,#], Yuping Wen[1,#], Zhen Xu[1], Haodong Lin[2,*], Guofeng Shao[3,4,*] and Xiajun Li[1,*,$]

[1]School of Life Science and Technology, ShanghaiTech University, Shanghai 201210, China

[2]Department of Orthopedic Surgery, Shanghai General Hospital, Shanghai Jiaotong University School of Medicine, 100 Haining Road, Shanghai 200080, China

[3]Department of Cardiovascular Surgery, The Affiliated Lihuili Hospital of Ningbo University, Ningbo City, Zhejiang 315041, China

[4]Ningbo Key Laboratory for Innovative Research in Aortic Surgery Diagnosis and Treatment, The Affiliated Lihuili Hospital of Ningbo University, Ningbo City, Zhejiang 315041, China

Supplemental Figure Legends

Figure S1. Three DNMT proteins maintained DNA methylation in three intergenic regions in the extraembryonic ectoderm of the post-implantation embryos. DNA methylation was analyzed for three intergenic regions (Intergenic 1, Intergenic 2 and Intergenic 3) in the trophectoderm (TE) of the wild-type (WT) E3.5 embryos, the extraembryonic ectoderm (EXE) of WT and *Dnmt* mutant E6.5 and E7.5 embryos. Vertical axis, % of DNA methylation. A) DNA methylation was analyzed for three intergenic regions

in the WT TE samples, the EXE samples of WT, *Dnmt1* mutant (*Dnmt1* KO), *Dnmt3* double mutant (*Dnmt3* DKO) and *Dnmt* triple mutant (*Dnmt* TKO) E6.5 embryos. B) DNA methylation was analyzed for three intergenic regions in the WT TE samples, the EXE samples of WT, *Dnmt1* mutant (*Dnmt1* KO), *Dnmt3* double mutant (*Dnmt3* DKO) and *Dnmt* triple mutant (*Dnmt* TKO) E7.5 embryos. DNA methylation levels in three intergenic regions were compared by using one-way ANOVA with Dunnett multiple comparison test. The obtained statistical analysis results are shown on the figures with a p-value or with the following symbols: *, $p<0.05$; **, $p< 0.01$; ***, $p<0.001$. ns, not statistically significant, with a p-value more than 0.1.

Figure S2. Three DNMT proteins maintained DNA methylation in the intergenic regions in the extraembryonic ectoderm of the post-implantation embryos according to the IGV plots. DNA methylation was analyzed for the individual CpG sites of three intergenic regions (Intergenic 1, Intergenic 2 and Intergenic 3) in the trophectoderm (TE) of the wild-type (WT) E3.5 embryos, the extraembryonic ectoderm (EXE) of WT and *Dnmt* mutant E6.5 and E7.5 embryos. The DNA methylation level is shown with the scale of 0-1 on the IGV plots. A) The DNA methylation IGV plots are shown for the individual CpG sites of Intergenic 1. B) The DNA methylation IGV plots are shown for the individual CpG sites of Intergenic 2. C) The DNA methylation IGV plots are shown for the individual CpG sites of Intergenic 3.

Figure S3. Three DNMT proteins were complementary in maintaining DNA methylation in the CpG island regions in the extraembryonic ectoderm of the post-implantation embryos. DNA methylation was analyzed for the CpG island (CGI) regions in the trophectoderm (TE) of the wild-type (WT) E3.5 embryos, the extraembryonic ectoderm (EXE) of WT and *Dnmt* mutant E6.5 and E7.5 embryos. Vertical axis, % of DNA methylation in the CGI regions. A) DNA methylation was examined for the CGIs in the WT TE samples, the EXE samples of WT, *Dnmt1* mutant (*Dnmt1* KO), *Dnmt3* double mutant (*Dnmt3* DKO)

and *Dnmt* triple mutant (*Dnmt* TKO) E6.5 embryos. B) DNA methylation was examined for the CGIs in the WT TE samples, the EXE samples of WT, *Dnmt1* mutant (*Dnmt1* KO), *Dnmt3* double mutant (*Dnmt3* DKO) and *Dnmt* triple mutant (*Dnmt* TKO) E7.5 embryos. Statistical analysis of DNA methylation in the CGIs was carried out by using one-way ANOVA with Dunnett multiple comparison test. The values on the figures are shown as follows: *, $p<0.05$; **, $p< 0.01$; ***, $p<0.001$. ns, not statistically significant.

Figure S4. Three DNMT proteins maintained DNA methylation at two pluripotent genes in the extraembryonic ectoderm of the post-implantation embryos. DNA methylation at two pluripotent genes (*Oct4* and *Nanog*) was analyzed for the genomic DNA samples derived from the trophectoderm (TE) of the wild-type (WT) E3.5 embryos, the extraembryonic ectoderm (EXE) of WT and *Dnmt* mutant E6.5 and E7.5 embryos. Vertical axis in Figure S4A-S4B, % of DNA methylation. Statistical analysis for DNA methylation in Figure S4A-S4B was carried out by using one-way ANOVA with Dunnett multiple comparison test. The DNA methylation level is shown with the scale of 0-1 on the IGV plots in Figure S4C-S4D. The values on the figures are shown as follows: *, $p<0.05$; **, $p< 0.01$; ***, $p<0.001$. ns, not statistically significant.

A) DNA methylation was examined for the trophectoderm (TE) of the wild-type (WT) E3.5 embryos, the extraembryonic ectoderm (EXE) of WT and *Dnmt* mutant E6.5 embryos.

B) DNA methylation was examined for the trophectoderm (TE) of the wild-type (WT) E3.5 embryos, the extraembryonic ectoderm (EXE) of WT and *Dnmt* mutant E7.5 embryos.

C) The DNA methylation IGV plots are shown for the individual CpG sites of the *Oct4* gene in the trophectoderm (TE) of the wild-type (WT) E3.5 embryos, the extraembryonic ectoderm (EXE) of WT and *Dnmt* mutant E6.5 and E7.5 embryos. TE-1 and TE-2, two different TE samples of WT E3.5 embryos.

D) The DNA methylation IGV plots are shown for the individual CpG sites of the *Nanog* gene in the trophectoderm (TE) of the wild-type (WT) E3.5 embryos, the extraembryonic ectoderm

(EXE) of WT and *Dnmt* mutant E6.5 and E7.5 embryos. TE-1 and TE-2, two different TE samples of WT E3.5 embryos.

Figure S5. Three DNMT proteins maintained DNA methylation in some lineage marker genes in the extraembryonic ectoderm of the post-implantation embryos. DNA methylation was examined for three lineage marker genes (*Gata4*, *Gata6* and *Cdx2*) in the trophectoderm (TE) of the wild-type (WT) E3.5 embryos, the extraembryonic ectoderm (EXE) of WT and *Dnmt* mutant E6.5 and E7.5 embryos. Vertical axis in Figure S5A-S5B, % of DNA methylation. Statistical analysis for DNA methylation in Figure S5A-S5B was carried out by using one-way ANOVA with Dunnett multiple comparison test. The DNA methylation level is shown with the scale of 0-1 on the IGV plots in Figures S5C-S5E. The values on the figures are shown as follows: *, $p<0.05$; **, $p< 0.01$; ***, $p<0.001$. ns, not statistically significant.

A) DNA methylation was examined for the trophectoderm (TE) of the wild-type (WT) E3.5 embryos, the extraembryonic ectoderm (EXE) of WT and *Dnmt* mutant E6.5 embryos.

B) DNA methylation was examined for the trophectoderm (TE) of the wild-type (WT) E3.5 embryos, the extraembryonic ectoderm (EXE) of WT and *Dnmt* mutant E7.5 embryos.

C) The DNA methylation IGV plots are shown for the individual CpG sites of the *Gata4* gene in the trophectoderm (TE) of the wild-type (WT) E3.5 embryos, the extraembryonic ectoderm (EXE) of WT and *Dnmt* mutant E6.5 and E7.5 embryos. TE-1 and TE-2, two different TE samples of WT E3.5 embryos.

D) The DNA methylation IGV plots are shown for the individual CpG sites of the *Gata6* gene in the trophectoderm (TE) of the wild-type (WT) E3.5 embryos, the extraembryonic ectoderm (EXE) of WT and *Dnmt* mutant E6.5 and E7.5 embryos. TE-1 and TE-2, two different TE samples of WT E3.5 embryos.

E) The DNA methylation IGV plots are shown for the individual CpG sites of the *Cdx2* gene in the trophectoderm (TE) of the wild-type (WT) E3.5 embryos, the extraembryonic ectoderm

(EXE) of WT and *Dnmt* mutant E6.5 and E7.5 embryos. TE-1 and TE-2, two different TE samples of WT E3.5 embryos.

Figure S6. DNA methylation was increased at some somatic DMRs in the imprinted regions in EXE after implantation although DNA methylation remained relatively stable at the corresponding ICRs in EXE of the post-implantation embryos. DNA methylation was examined for some somatic DMRs (sDMRs), which are located in the imprinted regions containing the ICRs with seemingly stable DNA methylation after implantation, in the trophectoderm (TE) of the wild-type (WT) E3.5 embryos, the extraembryonic ectoderm (EXE) of WT and *Dnmt* mutant E6.5 and E7.5 embryos. These sDMRs include the *Igf2r* TSS DMR in the *Igf2r* imprinted region, *Cdkn1c*, *Slc22a18*, *Kcnq1* Intergenic 1 and *Kcnq1* Intergenic 2 DMRs in the *Kcnq1ot1* imprinted region, and *Magel2*, *Ndn*, *Peg12* and *Mkrn3* DMRs in the *Snrpn* imprinted region. Horizontal axis, the analyzed DNA samples from TE of WT E3.5 embryos, and from EXE of WT, *Dnmt1* mutant (*Dnmt1* KO), *Dnmt3* double mutant (*Dnmt3* DKO) and *Dnmt* triple mutant (*Dnmt* TKO) E6.5 or E7.5 embryos. Vertical axis, % of DNA methylation. DNA methylation level was compared by using one-way ANOVA with Dunnett multiple comparison test. The statistical analysis results are shown on the figures as follows: *, $p<0.05$; **, $p< 0.01$; ***, $p<0.001$. ns, not statistically significant, with a p-value over 0.1. A) DNA methylation at these sDMRs in EXE of E6.5 embryos was compared with that in TE of E3.5 embryos.
B) DNA methylation at these sDMRs in EXE of E7.5 embryos in comparison to TE of E3.5 embryos.

Figure S7. Increased DNA methylation was observed in most somatic DMRs in EXE of post-implantation embryos that are located in the imprinted regions containing the ICRs with increased DNA methylation in EXE after implantation. DNA methylation was examined for some somatic DMRs (sDMRs) in the trophectoderm (TE) of the wild-type (WT) E3.5 embryos, the extraembryonic ectoderm (EXE) of WT and *Dnmt* mutant E6.5 and E7.5

embryos. These sDMRs include the *Zim2* and *Zim3* DMRs located in the *Peg3* imprinted region, *Zdbf2* DMR in the *Gpr1* imprinted region, *Nesp* DMR in the *Nespas* imprinted region, and *Igf2* DMR0, *Igf2* DMR1 and *Igf2* DMR2 in the *Igf2-H19* imprinted region. Horizontal axis, the analyzed DNA samples from TE of WT E3.5 embryos, and from EXE of WT, *Dnmt1* mutant (*Dnmt1* KO), *Dnmt3* double mutant (*Dnmt3* DKO) and *Dnmt* triple mutant (*Dnmt* TKO) E6.5 or E7.5 embryos. Vertical axis, % of DNA methylation. DNA methylation level was compared by using one-way ANOVA with Dunnett multiple comparison test. The statistical analysis results are shown on the figures as follows: *, p<0.05; **, p< 0.01; ***, p<0.001. ns, not statistically significant, with a p-value over 0.1. A) DNA methylation at these sDMRs in EXE of E6.5 embryos was compared with that in TE of E3.5 embryos, with increased DNA methylation in EXE of WT E6.5 embryos after implantation.

B) DNA methylation at these sDMRs in EXE of E7.5 embryos in comparison to TE of E3.5 embryos, with increased DNA methylation in EXE of WT E7.5 embryos after implantation.

C) DNA methylation at *Igf2* DMR0 in EXE of E6.5 embryos in comparison to TE of E3.5 embryos.

D) DNA methylation at *Igf2* DMR0 in EXE of E7.5 embryos compared with that in TE of E3.5 embryos.

Figure S8. DNMT3A and DNMT3B played important roles in de novo and maintenance DNA methylation in the somatic DMRs of *Dlk1-Meg3* imprinted region in the extraembryonic ectoderm of the post-implantation embryos. DNA methylation was examined for three somatic DMRs (*Meg3* DMR, *Dlk1* DMR, and *Rian* DMR) in the *Dlk1-Meg3* imprinted region in the trophectoderm (TE) of the wild-type (WT) E3.5 embryos, the extraembryonic ectoderm (EXE) of WT and *Dnmt* mutant E6.5 and E7.5 embryos. Horizontal axis, the analyzed DNA samples from TE of WT E3.5 embryos, and from EXE of WT, *Dnmt1* mutant (*Dnmt1* KO), *Dnmt3* double mutant (*Dnmt3* DKO) and *Dnmt* triple mutant (*Dnmt* TKO) E6.5 or E7.5 embryos. Vertical axis, % of DNA methylation. DNA methylation level was compared by using one-way ANOVA with Dunnett multiple comparison test. The

statistical analysis results are shown on the figures as follows: *, $p<0.05$; **, $p< 0.01$; ***, $p<0.001$. ns, not statistically significant, with a p-value over 0.1. A) DNA methylation at *Meg3* and *Dlk1* DMRs in EXE of E6.5 embryos in comparison to TE of E3.5 embryos. B) DNA methylation at *Meg3* and *Dlk1* DMRs in EXE of E7.5 embryos in comparison to TE of E3.5 embryos. C) DNA methylation at *Rian* DMR in EXE of E6.5 embryos compared with that in TE of E3.5 embryos. D) DNA methylation at *Rian* DMR in EXE of E7.5 embryos compared with that in TE of E3.5 embryos.
